# Insights into the regulation of intrinsically disordered proteins in the human proteome by analyzing sequence and gene expression data

**DOI:** 10.1186/gb-2009-10-5-r50

**Published:** 2009-05-11

**Authors:** Yvonne JK Edwards, Anna E Lobley, Melissa M Pentony, David T Jones

**Affiliations:** 1 Bioinformatics Group, Department of Computer Science, University College London, Gower Street, London, WC1E 6BT, UK

## Abstract

**Background:**

Disordered proteins need to be expressed to carry out specified functions; however, their accumulation in the cell can potentially cause major problems through protein misfolding and aggregation. Gene expression levels, mRNA decay rates, microRNA (miRNA) targeting and ubiquitination have critical roles in the degradation and disposal of human proteins and transcripts. Here, we describe a study examining these features to gain insights into the regulation of disordered proteins.

**Results:**

In comparison with ordered proteins, disordered proteins have a greater proportion of predicted ubiquitination sites. The transcripts encoding disordered proteins also have higher proportions of predicted miRNA target sites and higher mRNA decay rates, both of which are indicative of the observed lower gene expression levels. The results suggest that the disordered proteins and their transcripts are present in the cell at low levels and/or for a short time before being targeted for disposal. Surprisingly, we find that for a significant proportion of highly disordered proteins, all four of these trends are reversed. Predicted estimates for miRNA targets, ubiquitination and mRNA decay rate are low in the highly disordered proteins that are constitutively and/or highly expressed.

**Conclusions:**

Mechanisms are in place to protect the cell from these potentially dangerous proteins. The evidence suggests that the enrichment of signals for miRNA targeting and ubiquitination may help prevent the accumulation of disordered proteins in the cell. Our data also provide evidence for a mechanism by which a significant proportion of highly disordered proteins (with high expression levels) can escape rapid degradation to allow them to successfully carry out their function.

## Abstract 2

Signals for microRNA targeting and ubiquitination are enriched in intrinsically disordered proteins, but some highly disordered proteins can escape rapid degradation.

## Background

Natively unfolded or disordered proteins are proteins that do not form a stable three-dimensional structure in their native state. A disordered protein can be either completely unfolded or comprise both folded and unfolded segments [1-4]. Previous analyses have shown that the presence of large regions of disorder within proteins correlates strongly with function [1-20]. These functions typically relate to gene regulation and signaling classes that are of particular importance to higher organisms [6,21]. Previous work has also shown that over 30% of proteins in eukaryotic genomes are likely to be disordered, a percentage that is much higher than found within prokaryotic genomes [6,12,22,23]. Whilst there are functional benefits that accrue from disordered proteins, the use of disorder carries with it significant risks [24]. The prevalence of human diseases that correspond to highly disordered proteins is striking [24-31]; these include diabetes, neurodegenerative disorders [25-28], cardiovascular disease [29] and cancer [30]. In fact, many neurodegenerative disorders arise from the aggregation of disordered proteins [25-28]. If disordered proteins are indeed potential hazards to the healthy maintenance of human cells, then both their production and disposal should be very carefully regulated. Such is the danger of protein aggregation in living cells that a number of efficient degradation mechanisms are in place to quickly dispose of misfolded proteins [32]. The problem for disordered proteins may well be to survive long enough to carry out their function in such a hostile environment.

The equilibrium level of a protein depends on its rate of production relative to its rate of degradation. The quantity of a protein produced in the cell is affected by the expression level of its mRNA transcript. The levels of gene expression are controlled in the cell in a number of different ways - for example, by varying the rates of transcription and translation and altering the rate at which mRNA is degraded. In combination with transcription, mRNA degradation plays a critical role in regulating gene expression [33,34]. If proteins need to remain in the disordered state for any length of time, they need to either bypass the endogenous degradation pathways (such as the ATP-dependent proteolytic 26S proteasome [32]) that specifically target unfolded proteins or be produced in sufficient quantity to temporarily overload the protein degradation pathways. The second option is, of course, extremely risky as high production levels of disordered proteins may result in aggregation. This suggests that the first option is the most likely, but in this case, how can disordered proteins escape rapid degradation to allow them to successfully carry out their function.

Recent work suggested that disordered residues make a protein more susceptible to intracellular degradation [35]. The *in vivo *half-lives of yeast proteins were shown to correlate with disorder as opposed to the actual degradation signals and motifs. In our study we analyze biological properties known to regulate and affect the degradation rates of proteins and transcripts to investigate how these correlate with protein disorder. Gene expression is a continuous process spanning transcription factor activation, nuclear localization of transcription factors, chromatin decompaction, coupled initiation and 5' capping of transcripts, coupled transcription and mRNA processing, splicing, cleavage and 3' polyadenylation, mRNA packaging, mRNA export into the cytoplasm, translation and protein folding [36]. Biological processes that lower the mRNA copy numbers include proteolytic degradation by proteases, microRNA (miRNA):mRNA targeting and destruction of mRNA by nucleases. Here, we characterize absolute mRNA levels, mRNA decay rates, protein stability, predicted miRNA targeting and ubiquitination to assess whether disordered proteins (and their encoding transcripts) display any unusual characteristics.

miRNAs are a class of small non-coding RNA molecules (comprising about 22 nucleotides) that regulate gene expression and mediate diverse cellular processes such as development, differentiation, proliferation and apoptosis [37-41]. miRNAs target the 3' untranslated regions of mRNA molecules, which typically results in the down-regulation of gene expression by translational repression and/or a reduction of mRNA transcript levels [42]. Several algorithms are available to predict the mRNA targets [43-51].

Ubiquitination is a reversible post-translational modification of cellular proteins where ubiquitin (a 76 residue protein) is covalently attached to the ε amino group of lysines of target proteins. Diverse forms of ubiquitin modifications exist and influence the functional outcome of target proteins in distinct ways [52,53]. Mono-ubiquitination or multi-ubiquitination are implicated in various nonproteolytic cellular functions, including endocytosis, endosomal sorting and DNA repair [52]. Polyubiquitination is mainly associated with proteasomal degradation [54,55]. Whilst ubiquitination can determine the fate of a given protein for proteolytic degradation by the 26S proteosome, ubiquitination of transcription factors with a VP-16 activation domain is also shown to be required for transcriptional activation [56-58]. Like miRNA targeting [59-69], ubiquitination is crucial in regulating a variety of cellular processes in eukaryotes [59-61] and has significant implications in the etiology of a number of serious diseases such as cancer [62-64], neurodegeneration [65,66] and cardiovascular dysfunction [67-69].

To gain new insights into the regulation of disordered proteins, we carried out a series of studies to examine how a number of features known to affect protein and transcript degradation correlate with protein disorder. We investigated whether the mRNA transcripts encoding disordered proteins decay more rapidly. To establish mRNA expression patterns for transcripts encoding disordered proteins and to reveal novel insights into the molecular mechanisms of transcriptional regulation [70-74], mRNA expression levels were characterized in normal tissues and cell lines using public domain microarray expression datasets. Transcripts co-expressed with the transcripts encoding disordered proteins were identified to suggest the key biological pathways that are affected or under regulatory control of disordered proteins and their transcripts. We investigated whether disordered proteins have lower expression levels and/or the transcripts encoding them are more likely to be targeted by miRNA. One of the aims of this analysis was to use miRNA prediction to establish the trends that exist between possible miRNA targeting and the transcripts encoding disordered proteins. We examined if disordered proteins contain sites that are more susceptible to degradation using a novel ubiquitination site prediction tool. Protein turnover rates for disordered sequences were also investigated by considering stability determined from an *in vivo *study measuring protein turnover [75].

In this study, we examine the available human gene expression data and properties of the human proteome and transcriptome to investigate whether disordered proteins have any unusual characteristics in terms of their production and disposal in human cells. Specifically, we were interested in gaining insights into the means by which disordered proteins avoid early degradation without resorting to the severe risks of over-expression.

## Results

Five properties of the human proteins and transcripts were investigated in relation to disorder in the proteome. First, three expression profile studies on transcripts encoding disordered proteins were carried out: the general features of their expression levels were characterized; their expression profiles across the samples were clustered by abundance and functionally annotated to provide a classification of the biological roles of their encoded proteins; and transcripts co-expressed with them were identified. Second, we searched for correlation between the extent of mRNA decay rates and varying amounts of protein disorder encoded by transcripts. Third, the occurrence of disorder was compared with protein stability indices determined by a global stability profiling assay. Fourth, miRNA prediction tools were used to establish trends that exist between transcripts encoding disordered proteins and miRNA targeting. Finally, correlations between ubiquitination sites and protein disorder levels were investigated.

### Protein disorder and gene expression

#### Protein disorder and absolute gene expression levels

**Figure 1:**
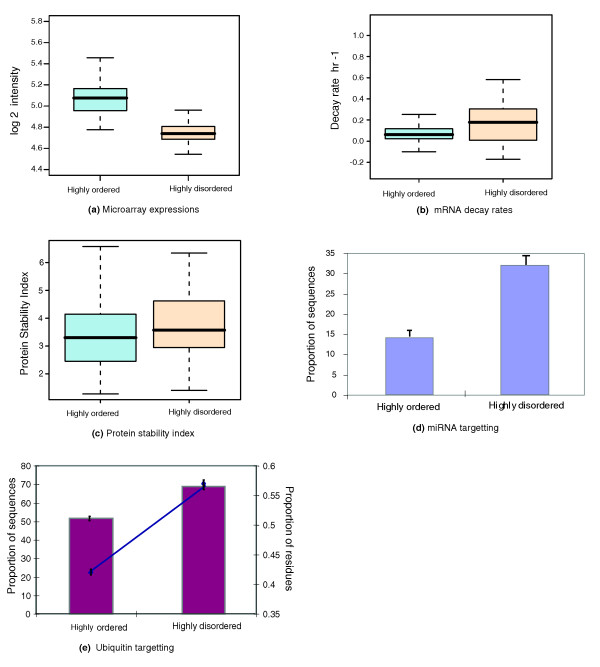
Properties of highly ordered and highly disordered proteins. **(a) **Box-plot distributions of the average expression levels for the transcripts encoding the highly ordered and the highly disordered proteins. **(b) **Box-plot of mRNA decay rates for the highly ordered and highly disordered proteins. **(c) **Box-plot of protein stability values. **(d) **The percentage of transcripts likely to be regulated by miRNA (y-axis) for the transcripts encoding the highly ordered and the highly disordered proteins. **(e) **The percentage of the proteins with one or more predicted ubiquitination sites (principal y-axis, burgundy bar chart) in the highly ordered and the highly disordered datasets; and the percentage of residues predicted as ubiquitination sites (secondary y-axis, navy line plot) versus different amounts of disorder.

On average, transcripts that encode highly disordered proteins are expressed in lower copy numbers than those that encode highly ordered proteins (Figure 1a). Figure 1a shows the average absolute gene expression values calculated across 207 normal tissue and cell line samples (Table 1). Whilst the scale for the absolute values is displayed in log_2 _units, in the decimal scale the absolute gene expression levels of the genes for transcripts that encode highly disordered proteins are roughly half those of the genes for transcripts that encode highly ordered proteins. A similar trend was obtained for transcripts that encode disordered and ordered proteins (Figure S1a in Additional data file 1).

**Table 1:** Bioinformatics analysis of expression of human genes across 207 samples from 75 different types of normal tissues and cell lines

Dataset	Description	Samples	Cel file sample replicates	References
[GEO:GSE1133]	Normal tissues and cell lines	144	72 × 2	[71]
[GEO:GSE2361]	Normal human tissues	36	36 × 1	[72]
[GEO:GSE2004]	Normal spleen	22	3 × 3 (spleen)	-
	liver and kidney		2 × 3 (liver)	
			1 × 3 (liver)	
			1 × 4 (kidney)	
[GEO:GSE781]	Normal kidney samples	5	1 × 5	[70]
Total		207	75	

**Figure 2:**
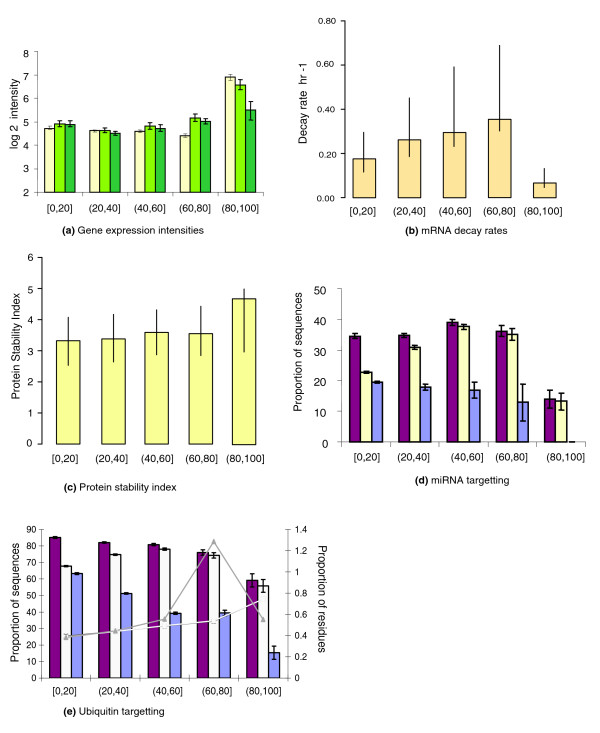
Correlation of features with percentage of disorder in the proteome. **(a) **Variation in absolute transcript expression as the percentage of disorder increases in the proteome (yellow bars). The bar charts represent the average sample expression for the groups of transcripts separated according to the percentage range (x-axis) of the total disordered residues in the encoded proteins. The y-axis scale represents log2 absolute expression. Expression levels for the transcripts with MF and BP GO terms at level 4 are shown as light green and dark green bars, respectively. **(b) **Variation of mRNA decay rate as disorder increases in the proteome. mRNA decay rates versus the percentage bins of disordered residues are shown. **(c) **Variation of protein stability as disorder increases in the proteome. The stability index versus the percentage bins of disordered residues are shown. **(d) **The proportion of protein coding transcripts targeted by miRNA (y-axis) as the percentage of disorder increases in the proteome. The datasets for the transcripts encoding the disordered proteins (burgundy) and ordered proteins (mauve) and the proteome (yellow) are shown. **(e) **The percentage of the proteins with one or more predicted ubiquitination sites against the percentage of disorder (principal y-axis, bar charts); and the percentage of residues predicted as ubiquitination sites against the percentage of disorder (secondary y-axis, line plots). The transcripts encoding the disordered proteins, the ordered proteins and the proteome are shown in burgundy, mauve and yellow (respectively).

To investigate whether these low expression levels were correlated with occurrence of disorder in the protein products, transcripts were grouped according to the frequency of disorder in the encoded protein (Figure 2a). As the percentage of disordered residues increases to between > 60% and ≤ 80% (or from now on (60,80]% in standard interval notation), the average gene expression level steadily decreases. However, for the (80,100]% disorder category the average sample expression levels were greater than expected using a Wilcoxon paired rank test (*P *< 0.0001). This (80,100]% category comprises <1% of the data (Table 2). To verify that these trends were independent of function, we filtered the data to impose equality of representation of biological process (BP) and molecular function (MF) Gene Ontology (GO) terms. Specifically, a maximum of ten randomly chosen examples were selected for each annotation term at specificity level 4 or below. The results (Figure 2a) indicate that the correlation between transcript expression levels and the amount of disorder are not dictated by function class bias and represent genuine and robust features of the data.

**Table 2:** Percentage of transcripts encoding disordered proteins predicted to be targeted by miRNA

	Total*	Unique^†^	Match^‡^	Percentage^§^
**Category of disorder**				
Highly disordered	877	827	257	31.08
Highly ordered	5,693	5,351	782	14.61
				
Disordered	15,095	14,282	5,056	35.40
Ordered	18,774	17,766	3,433	19.32
				
All proteins	33,869	32,010	8,468	26.45
				
**Percentage of disorder**				
Disordered				
[0,20]	4,271	4,055	1,402	34.57
(20,40]	6,957	6,603	2,300	34.83
(40,60]	3,036	2,866	1,119	39.04
(60,80]	679	644	233	36.18
(80,100]	152	143	20	13.99
Total	15,095	14,311	5,074	35.45
				
Ordered				
[0,20]	16,341	15,503	3,037	19.59
(20,40]	2,173	2,024	362	17.89
(40,60]	214	207	35	16.91
(60,80]	33	31	4	12.9
(80,100]	13	9	0	0
Total	18,774	17,774	3,438	19.34
				
Proteome				
[0,20]	20,612	19,536	4,429	22.67
(20,40]	9,130	8,618	2,658	30.84
(40,60]	3,250	3,073	1,154	37.55
(60,80]	712	675	237	35.11
(80,100]	165	152	20	13.16
Total	33,869	32,010	8,468	26.45

**Figure 3:**
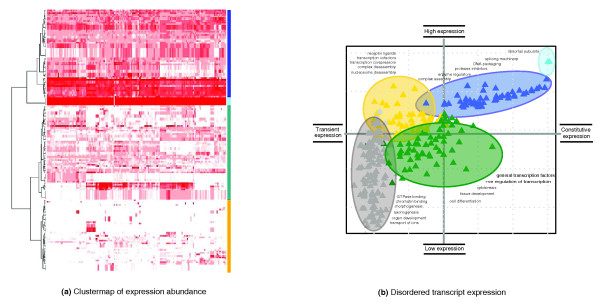
A summary of expression profiles for the highly disordered proteins. **(a) **The heat map displays four distinct transcript groups; constitutively expressed ribosomal subunits (light blue), high expressors (dark blue), medium expressors (green) and tissue specific expressors (gold). The clustering method was Ward's hierarchical clustering using Euclidean distances calculated over the absolute expression data matrix. Red colors indicate significantly high expression values (*P *< 0.001) within a sample tissue or cell line. **(b)**. Summary of expression-function trends for highly disordered transcripts. Log_10 _of the number of tissues in which the transcript is expressed (x-axis); log_10 _expression of the average magnitude of expression within each tissue (y-axis). The points have been jittered for overlap using a normally distributed noise value of 0.05 on the log_10 _scale.

For each data set, the *total number of transcripts encoding proteins and the ^†^number of unique protein sequences encoded by transcripts are given. ^‡^A match occurs when a transcript of a protein sequence matches an mRNA targeted by a miRNA. ^§^The percentage calculations are described in the Materials and methods. Values according to the category of disorder (Figures 1c, 2c) and the percentages of disordered residues (Figure 3c) are given.

#### Absolute gene expression profiles for highly disordered proteins

To differentiate modes of gene expression behavior among the highly disordered proteins, hierarchical clustering analysis of the absolute expression levels was carried out. The resulting heat map (Figure 3a) shows that the situation is not as simple as suggested in Figure 1. Five broad classes of expression patterns for the genes encoding highly disordered proteins could be defined (Figure 3; Tables S1 and S2 in Additional data file 2). These groups were functionally characterized by performing over-representation tests within each of the five classes. The first set of transcripts (light blue) encode proteins that are almost entirely disordered and contained within the (80,100]% disorder category. In this constitutively expressed group, all transcripts represent large ribosomal subunits that are essential parts of the transcription machinery and expressed in every cell. The second group (dark blue) represents transcripts that exhibit high expression levels in the majority of tissues and display little or no tissue specificity. The third group (green) contains transcripts expressed at medium levels. General DNA binding and transcription factor functions were over-represented in the proteins encoded by the medium expressor group. The fourth group (gold) contains transcripts expressed in a tissue-specific manner. The remaining transcripts form a group not detected to be abundant in any of the tissues studied and is referred to as the low or transient expressor group (gray). This low or transient expressor group comprises over 50% of transcripts analyzed (Table 3) and is primarily responsible for the low expression trend reported above. This suggests that over half of the transcripts encoding proteins with large regions of disorder are expressed either at transient or low levels.

**Figure 4:**
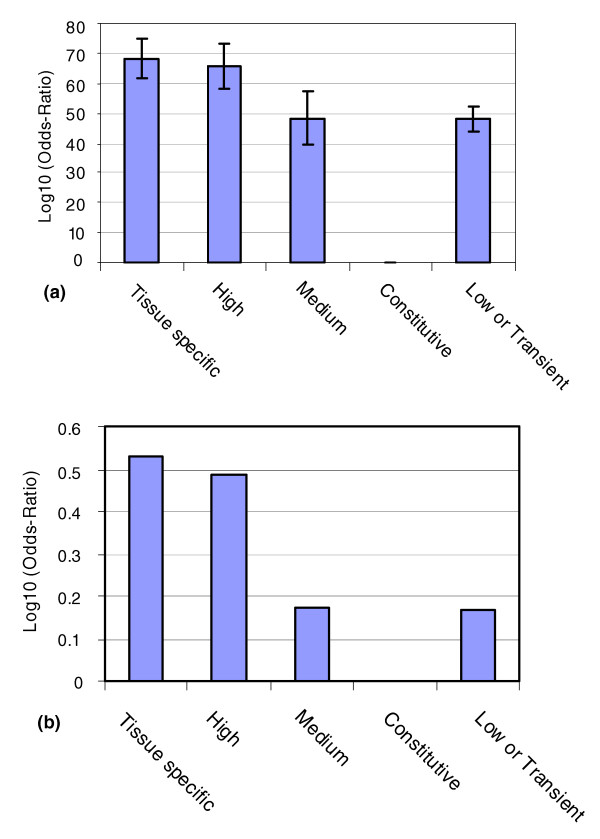
Summary of transcripts encoding highly disordered proteins as putative miRNA targets associated with expression profiles. **(a) **The percentage of the transcripts as predicted targets of miRNA (y-axis) versus the different datasets (x-axis) that comprise transcripts with different patterns of gene expression (Table 3). The error bars represent the confidence in the percent value according to different sample sizes for the different groups. **(b) **The log_10 _odds-ratio (y-axis) discriminates categories as under- and over-represented in relation to being a predicted miRNA target.

**Table 3:** miRNA targeting of disordered proteins with different gene expression profiles (Figure 4)

Expressor type	Total transcripts (frequency value)	Percentage of transcripts with different expression profiles	Transcripts with miRNA (frequency value)	Transcripts with no miRNA (frequency value)	Transcripts with miRNA (%)
Tissue specific	50 (47)	19.31	32	15	68.09
High	43 (41)	16.60	27	14	65.85
Medium	31 (31)	11.97	15	16	48.39
Constitutive	4 (1)	1.54	0	1	0
Transient or low	131 (129)	50.58	62	67	48.06
Total	259				

#### Co-regulated transcripts and the highly disordered proteins

A similar functional analysis was carried out for all transcripts detected to be significantly co-regulated with transcripts encoding disordered proteins. Co-regulation was established using significance of the correlation coefficient between transcripts and was calculated for transcript pairs in the (60,80]% and (80,100]% disorder groups. Using empirically derived *P*-values from the distribution of correlations, a significance threshold at either tail of *P *< 0.01 was used to describe transcripts as co-regulated. Several of the categories identified as enriched in the co-regulated transcript datasets overlapped and are summarized. In general, the activities of the ubiquitin degradation pathway and the proteolytic catabolic processes were observed to be anti-correlated (down-regulated) with the expression profiles of transcripts encoding highly disordered proteins. Functions enriched in the significantly correlated transcript set included protein complex formation, protein dimerization, protein homo-dimerization, protein hetero-oligomerization and enzyme inhibitors that reduce the activity of proteases (that is, enzymes catalyzing the hydrolysis of peptide bonds) (Table 4).

**Table 4:** Subsets of GO terms (biological process, molecular function and cellular component) over-represented for co-regulated transcripts encoding highly disordered proteins

Term	Description	Disorder (60,80]%	Disorder (80,100]%
[GO:0005769]	Early endosome	Down	Down
[GO:0005770]	Late endosome	Down	Down
[GO:0005838]	Proteasome regulatory particle	Down	Down
[GO:0016272]	Prefoldin complex	Down	
[GO:0031371]	Ubiquitin conjugating enzyme complex	Down	
[GO:0000145]	Exocyst	Down	
[GO:0000502]	Proteasome complex	Down	
[GO:0032991]	Macromolecular complex		Up
[GO:0043234]	Protein complex		Up
[GO:0019872]	Small conjugating protein ligase activity		Up
[GO:0042803]	Protein homodimerization activity		Up
[GO:0051131]	Chaperone-mediated protein complex assembly		Up
[GO:0008639]	Small protein conjugating enzyme activity		Up
[GO:0004842]	Ubiquitin-protein ligase activity		Up
[GO:0016874]	Ligase activity		Up
[GO:0006512]	Ubiquitin cycle		Up
[GO:0004869]	Cysteine protease inhibitor activity	Up	Up
[GO:0004866]	Endopeptidase inhibitor activity	Up	Up
[GO:0030414]	Protease inhibitor activity	Up	Up
[GO:0051082]	Unfolded protein binding	Up	Up
[GO:0046983]	Protein dimerization activity	Up	Up
[GO:0051291]	Protein hetero-oligomerization	Up	
[GO:0007032]	Endosome organization and biogenesis	Up	
[GO:0006983]	ER overload response	Up	
[GO:0051087]	Chaperone binding	Up	
[GO:0031579]	Lipid raft organization and biogenesis	Up	
[GO:0016235]	Aggresome	Up	
[GO:0016234]	Inclusion body	Up	
[GO:0016926]	Protein desumoylation	Up	
[GO:0008581]	Ubiquitin specific protease 5 activity	Up	
[GO:0006622]	Protein targeting to lysosome	Up	
[GO:0019783]	Small conjugating protein-specific protease activity		Down
[GO:0008219]	Cell death		Down
[GO:0007049]	Cell death		Down
[GO:0051603]	Proteolysis involved in cellular protein catabolic process	Down	Down
[GO:0004221]	Ubiquitin thiolesterase activity	Down	Down
[GO:0016197]	Endosome transport	Down	Down
[GO:0016874]	Ligase activity	Down	Down
[GO:0004843]	Ubiquitin-specific protease activity	Down	Down
[GO:0051082]	Unfolded protein binding	Down	Down
[GO:0000209]	Protein polyubiquitination	Down	Down
[GO:0006511]	Ubiquitin-dependent protein catabolic process	Down	
[GO:0006512]	Ubiquitin cycle	Down	
[GO:0051087]	Chaperone binding	Down	
[GO:0030968]	Unfolded protein response	Down	
[GO:0030100]	Regulation of endocytosis	Down	
[GO:0043488]	Regulation of mRNA stability	Down	
[GO:0031396]	Regulation of protein ubiquitination	Down	

Up, up-regulation; down, down-regulation.

### Protein disorder, mRNA decay rates and protein stability indices

The mRNA decay rates of the transcripts of 74 highly disordered proteins and 536 highly ordered proteins were compared. The mRNA decay rates for the transcripts encoding highly disordered proteins (0.190871 h^-1^) are more than twice that observed for the transcripts encoding highly ordered proteins (0.084944 h^-1^) (Figure 1b). A statistically significant difference (*P *< 0.02) between mRNA decay rates for transcripts encoding highly ordered and highly disordered proteins was found, with the highly disordered datasets having higher mRNA decay rates. The mRNA decay rates for the transcripts encoding 1,980 disordered proteins (0.177596 h^-1^) and 1,858 ordered proteins (0.096878 h^-1^) were also compared and a similar trend was obtained (Figure S1b in Additional data file 1).

We divided the 33,869 proteins into bins by percentage of disordered residues. When we compared the mRNA decay rates for each of the bins (Figure 2b), there was no significant difference between them. Although this result does not suggest that all disordered proteins show a significant association with higher mRNA decay rates, it does concur with our previous analysis of the (highly) ordered and (highly) disordered protein datasets, in showing a distinct difference between mRNA decay rates for both groups.

The protein stability measures of the highly disordered (179) and highly ordered groups (1,396) were also compared. We found a significant difference (*P *< 0.0005) between the half-lives of highly ordered and highly disordered proteins, with highly disordered proteins having longer half-lives (Figure 1c).

Consistent with our analysis of decay rates, we divided the 8,666 disordered proteins into bins by percentage of disordered residues. Protein stability indices showed no significant affiliation to a particular binned group, although the (80,100]% disorder bin showed much higher half-lives than the other binned groups (Figure 2c).

Since trends were observed between both mRNA decay rate and disorder, and protein half-life and disorder, the half-lives and decay rates were also compared to see if a relationship existed between mRNA decay rate and protein half-life. The Pearson correlation value between 1,446 overlapping sequences (-0.06) was not significant and suggested that these two characteristics are independent.

### Protein disorder and miRNA targets

Approximately one-quarter of protein coding transcripts are predicted miRNA targets (Table 2). The proportion of transcripts encoding highly disordered proteins that are likely to be miRNA targets is approximately twice that of transcripts encoding highly ordered proteins (Figure 1d; Table 2). The frequency of transcripts with at least one predicted miRNA target site is over-represented in the transcripts encoding highly disordered proteins (*P *< 0.003) and under-represented in the transcripts encoding highly ordered proteins (*P *< 0.00001) compared to all transcripts together (Figure S2a in Additional data file 1). A similar trend is observed when comparing the datasets of transcripts encoding ordered and disordered proteins (Table 2); the proportion of the transcripts encoding disordered proteins that are predicted as miRNA targets is approximately twice that of the transcripts encoding ordered proteins (Figure S1c in Additional data file 1; Table 2). miRNA targets are over-represented in the transcripts encoding disordered proteins (*P *< 0.00001) and under-represented in the transcripts encoding ordered proteins (*P *< 0.00001) compared to all transcripts together (Figure S2b in Additional data file 1).

For the transcripts encoding the proteome, the percent likely to be targeted by miRNA ranges between 13.2% and 37.6% (Figure 2d; Table 2). The percentage of transcripts regulated by miRNA increases (approximately 8%) with increasing percentage of protein disorder for the first three binned categories (Figure 2c; Table 2). The percent of predicted miRNA targets for transcripts remains high (35.1%) for the (60,80]% disorder category and low (13.2%) for the [80,100]% disorder category. Consistently, the likely miRNA targets are under-represented in the [0,20]% and (80,100]% disorder categories at *P *< 0.00004 (Figure S2c in Additional data file 1) and over-represented in the remaining three classes (*P *< 5.8 × 10^-7^; Figure S2c in Additional data file 1).

Similar trends are obtained using the PicTar (4-Way and 5-Way) software [43,46] (Figures 1d and 2d; Figure S1c in Additional data file 1). The trends were not observed using mirBase [51] and this could be because this prediction algorithm is reported to have a higher false positive rate than the other two programs (PicTar and TargetScanS) [47,49,50]. Redundancy in the datasets makes very little difference to the outcome (Table S3 in Additional data file 2). For example, the proteome and the protein sets filtered for redundancy have very similar percentages of transcripts predicted as targets of miRNA (Table 2; Table S3 in Additional data file 2).

We investigated the patterns of the predicted miRNA targets in the transcripts for disordered proteins in relation to the different expression profiles (Figures 3 and 4 and Table 3). The probes on the microarray chip have a higher representation of predicted miRNA targets (38%) in comparison with the transcriptome encoding the human proteome (26.45%) (Table 2). We compared the protein coding transcripts for the five datasets (Figure 3) using the probes on the microarray chip as a universal protein baseline. The data from the constitutive group had too few data points from which to make inferences (Table 3 and Figures 3 and 4). The tissue-specific expressors (gold) and the high expressors (dark blue) have high expression levels. The main difference between the two classes is that the tissue-specific expressors (gold) have high expression in one or few tissues (Figure 3) and the high expressors (dark blue) have high expression in almost all tissues (Figure 3). These two groups characterized by high levels of gene expression have high percentages of transcripts predicted as miRNA targets (68.09% and 65.85%, respectively; Table 3 and Figure 4). The medium expressors (green) and the low or transient expressors (white) with more moderate levels of gene expression have lower percentages of predicted miRNA targeting (48.39% and 48.06%, respectively). These results suggest that the transcripts of disordered proteins with high levels of expression are more likely to be regulated by miRNA compared to those with moderate and low or transient expression. In addition, the transcripts of highly disordered proteins belonging to the four expression profiles (tissue-specific, high expressors, medium expressors and low or transient expressors) are more likely to be miRNA targets than the transcripts on the microarray chip (Figure 4b). This observation supports the trend observed previously (Table 2) that transcripts encoding disordered proteins are more likely to be targeted by miRNAs compared to protein coding transcripts in general (Figure 4; Figures S1c and S2c in Additional data file 1).

### Protein disorder and ubiquitination

To our knowledge, this study presents the first estimate of the percentage of proteins of the human proteome with at least one predicted ubiquitination site and the percentage of residues predicted as ubiquitination sites. We predict that 70.71% of proteins have at least one ubiquitination site and 0.42% of amino acid residues in the proteome are ubiquitination sites.

The percentage of proteins predicted to contain at least one ubiquitination site and the percentage of residues predicted as ubiquitination sites are higher in disordered proteins compared to ordered proteins. Comparing the highly disordered proteins with the highly ordered proteins, we observe increases of 33.81% and 42.50% in the percentage of proteins possessing at least one ubiquitination site and the percentage of residues predicted to be ubiquitination sites, respectively (Figure 1e). The proteins possessing at least one ubiquitination site are slightly over-represented in the highly disordered proteins (*P *< 0.98; Figure S3a in Additional data file 1) and grossly under-represented in the highly ordered proteins (*P *< 2.2 × 10^-16^; Figure S3a in Additional data file 1). The first trend is not statistically significant. The predicted ubiquitination sites are over-represented in the highly disordered proteins (*P *< 2.2 × 10^-16^; Figure S4a in Additional data file 1) and under-represented for the highly ordered proteins (*P *< 0.002; Figure S4a in Additional data file 1). Comparing the disordered proteins with the ordered proteins, we observe increases of 33.57% and 12.8% in the percentage of proteins possessing at least one ubiquitination site and the percentage of residues predicted to be ubiquitination sites, respectively (Figure S1d in Additional data file 1). Proteins with one or more predicted ubiquitination sites are over-represented in the disordered datasets (*P *< 2.2 × 10^-16^; Figure S3b in Additional data file 1) and under-represented in the ordered proteins (*P *< 2.2 × 10^-16^; Figure S3b in Additional data file 1). A similar trend is obtained for the percentage of residues predicted as ubiquitination sites.

The relationship between the percentage of proteins with at least one ubiquitination site and the percentage of protein disorder is complex and non-linear, while the percentage of residues predicted as ubiquitination sites and the percentage of protein disorder are positively correlated. The percentage of proteins predicted to have a ubiquitination site increases with the percentage of protein disorder for the first three disorder categories (Figure 2e). The percentage of proteins predicted to have a ubiquitination site remains high at 74.3% for the (60,80]% disorder class and then drops significantly to 55.8% for the (80,100]% disorder category. This is consistent with proteins with one or more predicted ubiquitination sites being over-represented in the (20,40]%, (40,60]% and (60,80]% disorder categories (*P *< 0.04; Figure S3c in Additional data file 1) and under-represented in the [0,20]% and (80,100]% disorder categories (*P *< 0.00005; Figure S3c in Additional data file 1). On examination of the second ubiquitination descriptor, a different trend is observed; the percentage of residues predicted as ubiquitination sites increases as the percentage of protein disorder increases, illustrating a strong positive correlation between the two variables (Figure 2e). Proteins with one or more predicted ubiquitination sites are under-represented in the [0,20]% disorder category and over-represented in the remaining four disorder classes (*P *< 2.2 × 10^-16^; Figure S4c in Additional data file 1).

As lysine is over-represented in disordered regions [1,76,77], we investigated the percentage of residues predicted as ubiquitination sites in relation to the percentageof protein disorder, taking into account lysine residue biases (Figure S5a in Additional data file 1). First, we calculated a correlation coefficient for the percentage of predicted ubiquitination sites and the percentage of lysine composition for the five disorder categories and obtained a strong positive correlation (R = 0.844772). Second, we normalized the number of predicted ubiquitination sites with respect to the number of lysines for each dataset. The trends observed for the percentage of predicted ubiquitination sites normalized for lysine frequency and disorder are similar to those obtained with the percentage of predicted ubiquitin sites and disorder ignoring lysine biases (Figure S5b in Additional data file 1). Comparing the disorder categories with the order categories, the calculations normalized using the lysine frequency result in differences that are smaller in magnitude. For example, comparing the highly disordered proteins with the highly ordered proteins, an increase of 23.5% is observed instead of 42.5%, and comparing the disordered proteins with the ordered proteins, an increase of 4.4% is observed instead of 12.8%.

## Discussion

This is the first analysis presenting a comprehensive and systematic study of gene expression levels, mRNA decay rates, miRNA targeting and ubiquitination in association with transcripts encoding protein disorder in humans. Using the human proteome and transcriptome, we set out to elucidate novel insights into the regulation of disordered proteins. This aim was achieved and we discuss our findings in the following sections.

### Protein disorder and gene expression

On consideration of the gene expression levels for transcripts encoding disorder in proteins, two main trends emerge. Firstly, on average, the transcripts encoding disordered proteins are expressed to a significantly lower extent than those encoding ordered proteins. This suggests that, typically, the cell has evolved regulatory mechanisms to ensure that it does not have a large proportion of highly expressed transcripts encoding high amounts of protein disorder. Secondly, for the highly disordered proteins, there are five broad classes of gene expression patterns observed. These are constitutive expressors, high expressors, medium expressors, tissue-specific expressors and low or transient expressors. The constitutive expressors represent the transcripts that are constitutively and highly expressed across all samples. The high expressors contain protease inhibitors (specifically cysteine proteases) and enzyme regulatory functions. These proteins function in order to prevent their enzyme counterparts from cleaving peptide bonds; it is thus expected that their expression levels should remain relatively high in unstimulated tissue samples. This group is also enriched in functions directly related to splicing machinery, DNA packaging, nucleosome assembly and the components involved in DNA-protein and protein-protein complex assembly. In the medium expressor group, the transcription factors are predominantly positive regulators of transcription and are involved in muscle, cellular differentiation and general tissue development processes. The tissue-specific expressors are enriched in the transcription factors that target the nuclear hormone receptors and the ligands that are coordinately regulated with their receptor binding partners. The tissue-specific expressors are predominantly negative regulators of cell organization and promote complex disassembly and DNA unwinding and replication.

These novel observations have high biological relevance. Disordered proteins have important roles in the cell [1-31]; naturally they have to be expressed to carry out their specified function, but high levels of highly disordered proteins in the cell can cause a major problem through protein misfolding, misidentification and mis-signaling [24]. Our analysis suggests that one of the ways in which the cell keeps the level of highly disordered proteins under control is to keep the expression levels of transcripts encoding them low.

A recent study by Paliy and colleagues [78] claims that protein disorder is weakly positively correlated with gene expression in *Eschericha coli*. In our study, however, the trends between protein disorder and gene expression are complex and non-linear. Differences exist between the trends observed in *E. coli *[78] and those we report for human data. These differences are attributed to the differences in the methodologies (such as the disorder prediction methods and the definition of gene expression levels) and the consideration of species from disparate taxonomical classes. It is widely accepted that *E. coli *(a prokaryote) has about 5% of proteins that contain disordered regions whilst human (a eukaryote) has approximately 30% [6]. Paliy *et al*. [78] examined the highly expressed transcripts for proteins possessing high levels of predicted disorder in a prokaryote (*E. coli*). The types of genes that fall into this category encode RNA and protein chaperones, protein carriers, transcriptional and translational regulators and multi-enzyme complexes. Some of the genes are found only in prokaryotes - these include the peptidoglycan-associated lipoprotein and the glycine cleavage Complex H protein [78] - whilst other genes exist in all taxonomical classes and some of these are identified in our study (for example, the ribosomal proteins and the translational initiation factor). The products of these transcripts whilst highly disordered are required by all cell types and by all species (prokaryote or eukaryote) and the transcripts are constitutively expressed at high levels. In contrast to the *E. coli *study [78], we find distinct transcripts encoding highly disordered proteins that are low or transiently expressed. These proteins are predominantly transcription factors or activators of transcription involved in developmental processes specific to complex higher organisms (Figure 3).

### Protein disorder, mRNA decay rates and protein stability indices

We examined trends between mRNA decay rates and amounts of disordered residues, and between protein half-lives and frequency of disordered residues. The mRNA decay rates for the transcripts encoding highly disordered proteins are more than twice that observed for the transcripts encoding highly ordered proteins. Large quantities of proteins containing high levels of disordered residues can cause function problems for the cell [24] and a higher mRNA decay rate could indicate the necessity for removal of potentially problematic proteins. Our finding of a correlation between high mRNA degradation rates and disordered proteins would appear to be in agreement with this.

The correlation between increasing amounts of protein disorder and longer half-lives was not expected since intrinsically disordered sequences are known to be extremely susceptible to proteolytic degradation [79]. However, these results were consistent with findings reported in Yen *et al*. [75], who observed an enrichment of disorder promoting residues in more stable proteins. This may be a feature of the way in which stability was measured. Attachment of an amino-terminal GFPS tag to a protein in the global protein stability assay may interfere with cellular localization and the authors of this study recognize that the stability values tended only to be reliable for nuclear proteins. Additionally, it is likely that this tag affects correct folding of the protein and might obscure amino-terminal degradation signals (N-degrons), which are a major determinant of stability in eukaryotic sequences [80]. Considering these features of the available dataset, it may be that proteins with longer half-lives are enriched in the set that coincided with our data. However, the occurrence of highly stable sequences with long half-lives observed within sequences containing between 80% and 100% of disordered residues correlates well with the hypothesis that highly disordered proteins exist as complexes *in vivo*. A similar conclusion was drawn in the global protein stability profiling study where Yen *et al*. suggest that this mechanism constitutes a protection mechanism from cellular protein degradation machinery [75].

Recent work on protein disorder [81,82] arrived at similar conclusions to our study. They suggested that certain disordered proteins may be required to remain in the cell for long periods of time, and thus need to avoid the degradation process. They suggest that such avoidances are evident by an increase in protein stability for some disordered proteins. Separately, they also found a correlation between decay rates and mRNA stability for disordered proteins, in agreement with our analysis.

### Protein disorder and miRNA targets

We find a significantly higher level of predicted miRNA regulation of the transcripts encoding highly disordered proteins compared with the transcripts encoding highly ordered proteins. The predicted levels of miRNA regulation of the transcripts encoding highly disordered proteins are twice that observed for the transcripts encoding highly ordered proteins. Over one-third of the transcripts encoding disordered proteins are predicted to be regulated by miRNA. One-fifth of the transcripts encoding ordered proteins are predicted to be miRNA targets. Furthermore, miRNA-regulated gene expression is over-represented in transcripts encoding disordered proteins and under-represented in the transcripts coding for ordered proteins. These trends take into account 99% of the transcriptome encoding disordered proteins (that is, excluding the (80,100]% disorder category). We find that the transcripts of highly disordered proteins with high levels of expression are more likely to be affected by miRNA compared to those with moderate and low or transient expression. We provide strong evidence for miRNA regulation being particularly important for transcripts encoding disordered proteins. The observations make sense in a biological context. Typically, if a protein has high proportions of disorder, it is rapidly degraded in the cell [32]. For the cell, it would make economic sense to have an analogous system in place to handle the flagging up and degradation of the corresponding mRNA at the transcriptome level. This increased likelihood of miRNA binding to mRNA molecules that encode disordered proteins would regulate the gene expression of the mRNA molecule and prevent undesirable and wasteful translation of proteins no longer required by the cell.

### Protein disorder and ubiquitination

The percentage of residues predicted as possible ubiquitination sites increases with increasing amounts of disorder. Interestingly, the relationship between these two properties is linear and positive. The trend between the percentage of proteins predicted to have one or more ubiquitination sites and disorder is more complex. The (80,100]% category has the lowest proportion of proteins predicted to have one or more ubiquitination sites compared to the remaining four categories. This follows a similar trend observed for mRNA decay rates and predicted miRNA targeting. This suggests that a significantly lower proportion of these proteins are likely not to be down-regulated by ubiquitination. This supports the observation described earlier that a significant proportion of highly disordered proteins is required to be expressed at high levels in all tissues or some tissues, and some are sometimes constitutively expressed. If high proportions of these highly disordered proteins and their corresponding transcripts did have positive signals for targeted degradation, this could adversely affect fitness. Additionally, highly disordered proteins with one or more predicted ubiquitination sites that are not constitutively or highly expressed may have a higher chance of being removed from the cell, as they are likely to have a higher density of ubiquitination sites. The (80,100]% disorder category is most likely to have the highest density of ubiquitination sites (Figure 2e).

### Protein disorder in relation to the five properties studied

The increase in the decay rate of the transcripts encoding disordered proteins is likely attributable, in part, to the increase in predicted miRNA regulation. The transcripts encoding disordered proteins are targeted to a higher extent by miRNA compared to the transcripts encoding ordered proteins. This will result in the down-regulation of gene expression. The absolute gene expression levels and the predicted miRNA regulation are anti-correlated. The overall decrease in the gene expression of the transcripts encoding disordered proteins is likely attributable, in part, to the increased miRNA targeting that results in the down-regulation of these transcripts.

On the one hand, the majority of the disordered proteins have evolved with higher mRNA decay rates, higher levels of miRNA targeting, and higher levels of ubiquitination, which overall result in lower gene expression levels and protein levels for a high proportion of these disordered proteins compared to the ordered proteins. On the other hand, it is shown that for a significant proportion of highly disordered proteins, the converse is true. For the (80,100]% disorder class there is a decrease in mRNA decay rates, lower proportions of miRNA targeting and lower proportions of proteins being targeting for ubiquitination. These properties play a role in the high levels of gene expression observed in the highly disordered proteins compared to proteins with less disorder. The regulation of disordered proteins is affected by the various factors studied, and the relationships between these properties and protein disorder are inter-related, non-linear and complex.

Chen *et al*. [83] performed a structural biology analysis for the purpose of studying associations between structural vulnerability and co-expression in yeast and human. They claim that structural vulnerability (structural disorder) affects gene co-expression in a quantifiable manner [83]. In their study, they consider post-transcriptional regulation of transcripts of highly vulnerable proteins and find that 45% of human genes are predicted to have a least one miRNA target site compared to 82.9% of extremely vulnerable genes (87 out of 105) [83]. The mean number of miRNA target sites is 2.66 for human genes and 6.01 for vulnerable genes [83]. They show that vulnerability (disorder) requires significant additional regulation at the post-transcriptional level. This is an observation also made in our study; however, our miRNA study provides a more comprehensive analysis. For example, we investigate three different views of disorder. The first definition divides the transcriptome into transcripts encoding disordered proteins or ordered proteins. Second, transcripts encoding highly disordered or highly ordered proteins are considered. Third, we examined miRNA targeting of mRNAs encoding proteins with different percentages of disordered residues. Our study therefore provides more information in relation to disordered and highly disordered proteins. For example, we find that transcripts encoding disordered proteins are more likely to be targeted by miRNA than the transcripts encoding highly disordered proteins (Figures 1d and 2d; Figure S1c in Additional data file 1). Additionally, the method and the materials of Chen *et al*. [83] provide an estimate of miRNA targeting higher than our analysis and higher than estimates in other studies [39,45]. Our overall estimate of 26.5% for miRNA targeting agrees well with other estimates for miRNA targeting of protein coding transcripts [39,45]. miRNAs are shown to preferentially target genes with high transcriptional regulation complexity [84], and those involved in cellular signaling [85] and protein-protein interaction networks [86]. Functional properties such as transcriptional regulation, signaling and protein-protein interactions are associated with disordered proteins [1,2,5-9,78,83]. Our study highlights the association between predicted miRNA targets and protein disorder in a general way.

Mono-ubiquitination rather than polyubiquitination is the prevalent signal in intranuclear trafficking and triggers the first step of endocytosis [53,87-89]. The transcripts relating to polyubiquitination GO categories are down-co-regulated with highly disordered proteins (Table 4). Our results show that expression levels for the transcripts encoding highly disordered proteins are anti-correlated with transcripts involved in proteolysis and ubiquitin-dependent cellular catabolism. The expression levels for the transcripts encoding highly disordered proteins are positively correlated with proteolytic inhibitors. Whilst post-translational modification of proteins by ubiquitin is a key regulatory event, de-ubiquitination proteases, the enzymes that remove and process ubiquitin from proteins, are known to be functionally important [90]. Many of the de-ubiquitination proteases are cysteine proteases [90]. Transcripts coding for cysteine protease inhibitors, endopeptidase inhibitors and protease inhibitors are up-co-regulated with highly disordered protein transcripts (Table 4), which may suggest that protease enzymes involved in de-ubiquitination are not expressed when the expression of disordered protein transcripts is high. We hypothesize that if a highly disordered protein has a ubiquitin tag, the function of this tag is more likely to be brought to fruition [87,90]. Highly disordered proteins with one or more predicted ubiquitination sites that are not constitutively or highly expressed may have a higher chance of being removed from the cell, as they are likely to have a higher density of ubiquitination sites. The (80,100]% disorder category is likely to have the highest density of ubiquitination sites (Figure 2e). Since ubiquitination is a feature of many biological processes [87-89] the presence of these ubiquitin target sites may also be implicated in protein transport between membrane components, possibly serving as a sorting signal and/or a regulatory signal for internalization into the endocytic pathways.

Transcripts associated with GO terms involved with protein complex formation are co-expressed with transcripts of highly disordered proteins (Table 4). Transcripts belonging to the (60,80]% disorder class are co-expressed with transcripts involved in heterodimeric complex assembly whilst those that belong to the (80,100]% class are co-expressed with those transcripts involved in homodimeric complex assembly. Several disordered proteins are known to be involved in protein cellular complexes [5,78,83], providing support for this finding. Transcriptome and interactome studies are known to provide complementary results [91,92]. The results indicating that chaperone binding correlates with highly disordered proteins (Table 4) are not conclusive and this is supported by a recent analysis [93]; it is hypothesized that disordered proteins that bind chaperones do so to avoid aggregation and assist in complex assembly [94-97]. Expression levels for transcripts involved in catabolic processes are down co-regulated with expression of transcripts encoding disordered proteins, that is expression levels of these transcript groups are simultaneously low (Table 4). This suggests that there is a reduction in the catabolism of biopolymers (such as proteins) and that the transcripts encoding highly disordered proteins and the resulting protein product remain in the cell longer to carry out function. The gene expression measurements were taken from 200 normal, 'resting' and un-stimulated tissues and this could partly explain the down-regulation of catabolic processes as well as other observations.

## Conclusions

Our results suggest that the enrichment of miRNA targeting signals and ubiquitination signals may help prevent the accumulation of disordered proteins and their transcripts in the cell. Unexpectedly, for a proportion of highly disordered proteins, all four of these trends were reversed. The highly disordered proteins that are constitutively and/or highly expressed are shown to have low levels of estimated miRNA targeting, ubiquitination and lower mRNA decay rates, suggesting mechanisms by which highly disordered proteins can escape rapid degradation to allow them to successfully carry out their function. We conclude that the results of our study can serve as a baseline for characterizing the steady state abundance of disordered transcripts in normal tissues and cell lines as well as providing insights into how disordered transcripts might be regulated. These results can be used in future to compare disease states to normal states to identify disordered proteins, transcripts and modes of regulation that could be targets for therapeutic intervention in disease. Our study provides a better understanding of the regulation of transcripts for disordered proteins and some insights into the cellular regulatory mechanisms of key proteins that are likely to be involved in disease states.

## Materials and methods

### Disorder prediction in the human proteome

Human protein sequences were obtained from the Ensembl FTP website (Assembly version 35). Disorder predictions were carried out using DISOPRED2 [6] with a 2% false positive rate. Our definition of a disordered protein states that it must contain at least one region of 30 contiguous disordered residues. This cut-off was based on previous work [98]. Two or more disordered regions separated by a number of ordered residues were considered distinct disordered regions. Using MEMSAT3 [99] and PFILT [100], we filtered our datasets for transmembrane and coiled-coil regions. This ensured a low false positive rate in our disorder predictions. An ordered protein is defined as one that has no disordered regions; that is, it does not contain a contiguous region comprising 30 or more predicted disordered residues. Using these definitions [98], proteins were classed as being ordered or disordered (Table 2).

A second classification scheme was devised to generate protein datasets reflective of high order and high disorder. To achieve this, we divided the proteome into 3 groups: those containing less than 10 residues of disorder; those containing 60% or more disordered residues; and those not fitting within either group. For the highly ordered protein dataset (Table 2), we chose a lower end cut-off of less than ten residues of disorder to allow for false positive disordered residues. For the highly disordered proteins (Table 2), an upper cut-off of 60% was used to ensure that this group contained genuine high levels of disorder in proteins. A third scheme to classify disorder in proteins was based on the percentage of disordered residues in the protein. The percentage of disordered residues in the protein datasets were binned (Table 2). The first bin [0,20]% has from 0% (inclusive) to 20% (inclusive) residues disordered; the second bin (20,40]% has >20% and ≤40% residues disordered and so on until the final bin (80,100]%, which has >80% and ≤100% residues disordered.

### Protein disorder and gene expression

#### Microarray data pre-processing, normalization and summarization

We combined and integrated 207 normal tissue and cell line samples at the probe-level from microarray datasets (Table 1) downloaded from the Gene Expression Omnibus (GEO) database [101]. The Novartis gene expression atlas includes 79 samples, each having two replicates. Seven samples (cancer tissues and an unknown tissue type) were excluded (Table 1). Each experimental sample was adjusted for background using the GCRMA algorithm [102] and quantile normalized using a common reference distribution constructed from 50 selected and maximally varying U133a chip samples. Summarization was carried out on a per-transcript level using the Ensembl transcript hs133ahsenst, hs133ahsenstcdf and hs133ahsenstprobe custom cdf environments [103]. Sample R code [104] for the procedure can be found online [105].

#### Comparison of absolute transcript abundance

The transcripts identified on the microarray were divided into two distinct groups: those encoding highly disordered proteins (346) and those encoding highly ordered proteins (2,127). The average transcript abundance in each tissue was calculated and compared between the two groups. The same was carried out for disordered (10,681) and ordered (4,883) categories.

#### Evaluating significant absolute transcript expressions

Significance values were calculated for absolute transcript abundances within sample tissues and cell lines using Z scores to identify outliers. The null hypothesis that the transcript was not significantly expressed in the sample was rejected at *P*-values of < 0.05.

#### Evaluating significant expression correlations

Relative transcript abundances were obtained by double mean centering the absolute transcript levels across the tissues and between the probe-sets. The mean expression for each probe-set was weighted according to the number of replicates in each tissue group. Weights were calculated such that the contribution of sample replicates for the same tissue or cell line summed to 1. Co-expression of the transcript pairs was then evaluated using a weighted correlation coefficient. Significance of correlation was evaluated explicitly by applying a Z score transform to the distribution of Pearson correlation coefficients. Transcripts were considered to be significantly correlated and consequently co-expressed at *P*-values of < 0.01. This significance threshold corresponded to Pearson's correlation values of 0.749 and -0.729 at the upper and lower distribution tails, respectively (Figure S6 in Additional data file 1).

#### Gene Ontology analysis

GO annotations were downloaded from Ensembl BioMart [106] for BP and MF transcripts. To robustly identify over-representation of functions in the sets of transcripts co-regulated with disordered transcripts, a two step statistical testing procedure was used. First, multiple hypergeometric testing was performed to identify functions enriched within a set of co-regulated transcripts for a given disordered transcript. Prior probabilities for each function class were determined by the observed frequencies across all transcripts. The null hypothesis was rejected at *P*-values below 0.05 controlling the false discovery rate at 5%. Subsequently binomial testing was performed (assuming replacement) to model the enrichment of functions common to groups of co-regulated transcripts. The prior probability of enrichment for a function class between multiple sets of co-regulated transcripts was determined by evaluating the frequency of positive outcomes resulting from the hypergeometric test over all co-regulated transcripts. The false discovery rate for group-wise functional enrichment was controlled at 1%.

### Protein disorder, mRNA decay rates and protein stability indices

In previous work, Yang *et al*. [33] measured the mRNA decay rates of 5,245 human transcripts. The mapping of the EMBL (GenBank/DBJ) identifiers for these mRNA transcripts [33] to the Ensembl protein identifiers of our protein datasets was facilitated by the use of Biomart [106]. Using their EMBL (GenBank/DBJ) identifiers, we mapped the decay rates to the Ensembl protein identifiers of our dataset of human proteins. This gave a dataset of 3,839 proteins, each of which has an associated experimentally determined mRNA decay rate. We separated our 3,839 protein dataset into 3 groups; highly disordered (74 proteins); highly ordered (536 proteins); and the remainder (3,229 proteins).

Recent work by Yen *et al*. [75] reported half-life protein stability measures for more than 8,000 human proteins using their global protein stability assay. This study is one of the most comprehensive studies of *in vivo *stability measured for proteins in complex cellular mixtures. We mapped these stability measures to our disorder protein dataset, which resulted in a set of 6,886 proteins. We separated this dataset into three groups, as described above. This resulted in 179 highly disordered proteins, 1,396 highly ordered proteins and 5,311 remaining proteins.

### Protein disorder and miRNA targets

The predicted mRNA targets of mammalian miRNAs were downloaded from the TargetScanS website [44]. The dataset downloaded (release 4.2; April 2008) contains 218,298 mRNA target predictions. The predictions were performed using TargetScanS [44]. From this dataset, 7,928 unique genes (HUGO identifiers provided) were predicted to be targets of miRNA. The HUGO identifiers [107] for the mRNA genes were extracted into a gene list. An analysis pipeline was developed to establish whether a correlation exists between the disordered proteins and the transcripts targeted by miRNA. The first part uses the Ensembl database and the PERL API [108] to map the HUGO identifiers to Ensembl identifiers (the gene, the transcript and the protein) and extract the associated translated protein sequences. We identified 15,954 transcripts for the 7,928 HUGO gene identifiers. The second part categorizes the protein datasets based on the amounts of disorder (Table 2). With each protein category, a protein list is derived and protein sequence dataset is created. The third part compares each protein dataset with the translated products of the mRNA targets regulated by miRNAs by tallying identical protein sequences derived from the 15,954 transcripts and the transcripts from each disorder category. For a given dataset (Table 2), to calculate the percentage of protein transcripts that are predicted miRNA targets, the number of matches between the two datasets was divided by the total number of proteins in the disordered protein category under consideration and this fraction was multiplied by 100. Fisher's exact tests were carried out in R [109] to identify groups of transcripts that were enriched in miRNA target sites.

### Protein disorder and ubiquitination

Ubiquitin targeting signal sites were predicted for the peptides encoded by the Ensembl transcripts using a neural network-based predictor. Our predictor uses a single hidden-layer back-propagation network trained to recognize features of ubiquitin targeting signals over a sliding window of 21 amino acids in the target sequence. The network was trained using a balanced dataset of Swiss-Prot annotated ubiquitination sites [110] and rigorously cross-validated using a jack-knife leave-one-out approach. The performance of our predictor on both the Swiss-Prot training data and a non-redundant experimentally defined dataset [110] was also determined (Figure S7 in Additional data file1). Predictions were only reported at a false discovery rate of <5% estimated from our model. Although no ubiquitination predictors were available when we carried out this study, the Ubi-pred tool [111] has subsequently been published. This tool obtains a final area under the curve (AUC) [112] of 0.85, sensitivity of 70.86, specificity of 0.954 and a Matthews correlation coefficient of 0.69 compared to our own ubiquitination predictor, which obtains a final AUC of 0.88, sensitivity of 83.44, specificity of 85.43 and a Matthews correlation coefficient of 0.69. These statistics provided the justification for using our own ubiquitination prediction tool over Ubi-pred [111] due to the observed lower false positive rate. The percentage accuracy is defined here as 100% - Percentage of errors and the AUC is the area under the receiver operating characteristic (ROC) curve (a standard score for prediction algorithms between 0 and 1).

As an independent test of our ubiquitin site prediction algorithm, we scanned a dataset of putative and experimentally determined ligase target sequences [113] for the presence of ubiquitin modification sites (Table S4 in Additional data file 2). In total, 72.6% of sequences (257 of 345) were predicted to contain at least 1 modification site. This demonstrates that our *in silico *predictions are in good agreement with independent experimental outcomes.

## Abbreviations

AUC: area under the curve; BP: biological process; GEO: Gene Expression Omnibus; GO: Gene Ontology; MF: molecular function; miRNA: microRNA.

## Authors' contributions

YKE carried out the miRNA and ubiquitin target analysis and drafted the major part of the manuscript. AEL carried out the microarray analysis, ubiquitin predictions and function analysis and assisted in all other aspects of the work. MP carried out the analysis of decay rates and protein stability. DTJ provided the ubiquitin site prediction algorithm. DTJ and AEL conceived of the study and participated in its design and coordination. All authors contributed to and approved the final manuscript.

## Additional data files

The following additional data are available with the online version of this paper: supplementary Figures S1 to S7 (Additional data file 1); supplementary Tables S1 to S4 (Additional data file 2).

## Supplementary Material

Additional data file 1 Figure S1 is a four part figure detailing the distributions of four properties, expression abundance, decay rate, and frequency of miRNA and ubiquitin target sites between disordered and ordered sequences. The plots are similar to those shown in Figure 1 but use alternative definitions of disorder and order to partition the data. Figure S2 is a plot of the occurrence of miRNA target sites as the amount of disorder increases. Figure S2a represents the occurrence of miRNA target sites in highly ordered and highly disordered sequences. Figure S2b represents the occurrence of miRNA target sites between ordered and disordered sequences and Figure S2c shows the occurrence of miRNA target sites as the amount of disorder increases. Figure S3 is a series of plots showing the frequency of sequences that are predicted to contain at least one ubiquitin target site. Figure S3a compares these frequencies between highly ordered and highly disordered sequences. Figure S3b is a similar plot between ordered and disordered sequences. Figure S3c is a bar plot of the frequency of ubiquitinated sequences in populations of disordered, ordered and all sequences as the amount of disorder increases. Figure S4 is a series of plots showing the frequency of predicted ubiquitin target sites in relation to varying amounts of disorder. Figure S4a is a bar plot of the frequency of ubiquitinated residues in highly disordered and highly ordered sequences. Figure S4b is a bar plot of the occurrence of ubiquitinated residues between ordered and disordered sequences. Figure S4c is a plot of the frequency of ubiquitinated residues in ordered, disordered and all sequences as the proportion of disordered residues increases. Figure S5a, b provides evidence that the predictions of ubiquitin target sites are independent of the proportion of lysine residues in the sequence despite the fact that both increase with the amount of disorder in the sequence. Figure S5a is a plot of the relationship between predicted ubiquitin target sites and occurrence of lysine residues with increasing amounts of disorder. Figure S5b shows the occurrence of predicted ubiquitin target sites normalized to the frequency of lysine residues as disorder increases. Figure S6 shows the distribution of transcript pair correlations obtained from the samples in the combined microarray studies. The distribution was used to empirically derive *P*-value cut-offs for significant correlation values. Figure S7 is a receiver operating characteristic (ROC) curve obtained using the ubiquitin site prediction algorithm on experimentally determined ubiquitin target sites. Click here for file

Additional data file 2 Table S1 is a color coded listing of molecular function GO terms that are over-represented in clusters of disordered transcripts. Table S2 is a color coded listing of biological process GO terms that are over-represented in clusters of disordered transcripts. Tables S3a details the datasets used in the study and their composition when filtered for redundancy at 90% sequence identity. Table S3b lists the number of sequences that are classed as ordered and disordered when binned according to the amount of disorder present. Table S4 lists the ubiquitin target site predictions for the experimentally determined ligase target dataset. Click here for file
