# Supplementary material for: Insights into the regulation of intrinsically disordered proteins in the human proteome by analyzing sequence and gene expression data
Source: Genome Biol. 2009 May 11;10(5):R50. doi: 10.1186/gb-2009-10-5-r50 (PMC2718516; doi:10.1186/gb-2009-10-5-r50)

**Figure S1**. **Properties of ordered and disordered proteins**. (**a**) The box-plot distributions of the average expression levels for the transcripts encoding the ordered and disordered proteins. (**b**) A box-plot of mRNA decay rates for the ordered and the disordered proteins. (**c**) The percentage of transcripts likely to be regulated by miRNA (y-axis) for the transcripts encoding ordered and disordered proteins. (**d**) The percentage of the proteins with one or more predicted ubiquitination sites (principal y-axis, burgundy bar chart) in the ordered, and the disordered datasets. The percentage of residues predicted as ubiquitination sites (secondary y-axis, navy line plot) in the ordered and disordered proteins.

| **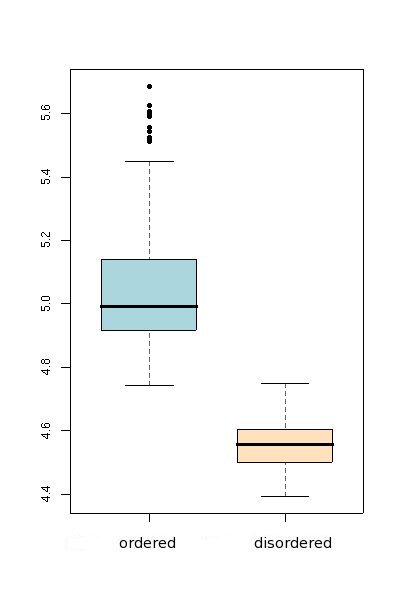** | **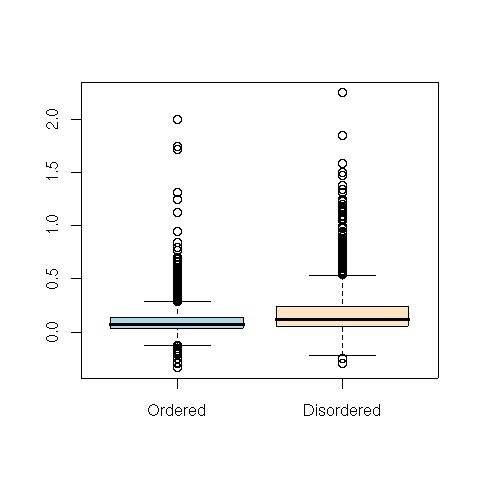** |
| --- | --- |
| **(a) Gene expression levels** | **(b) mRNA decay rates** |
| **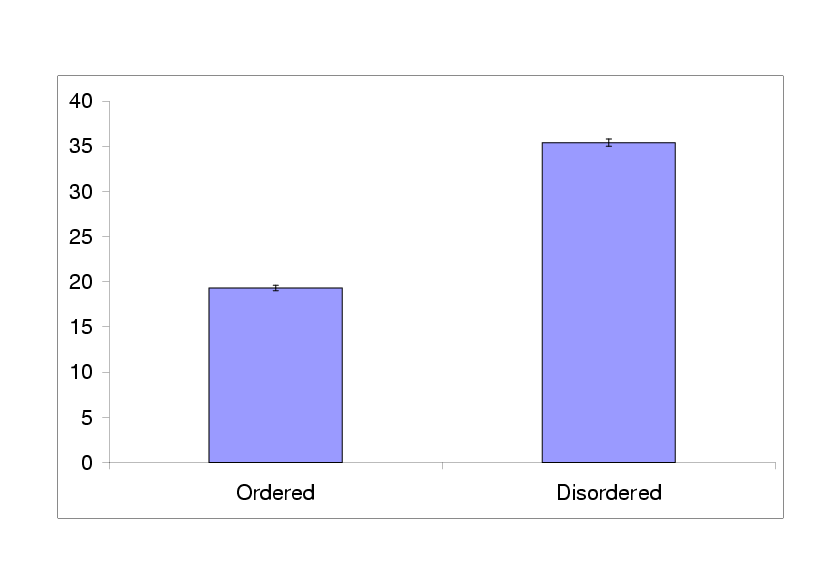** | **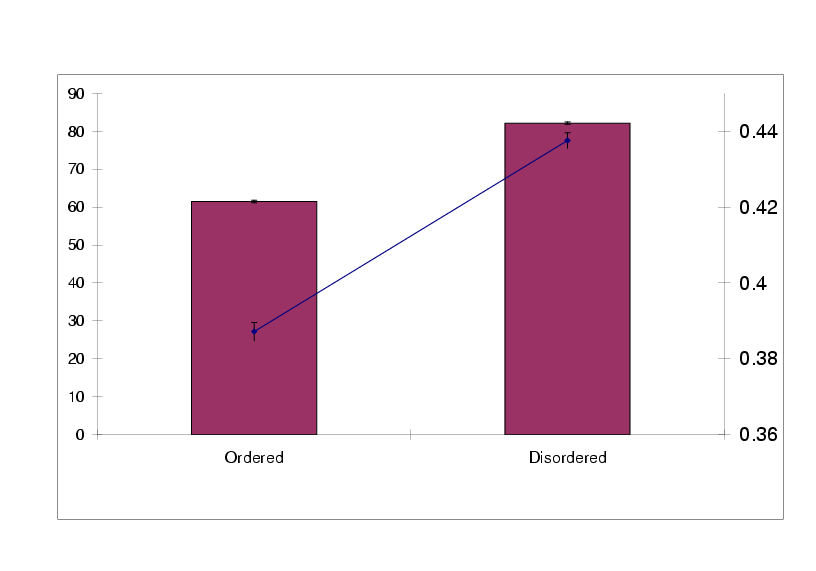** |
| **(c) miRNA** | **(d) Ubiquitination** |

**Figure S2**. **Biases in miRNA target prediction with increase in protein disorder**. For miRNA targets, the logs odd-ratio (y-axis) discriminate categories as under and over represented in protein datasets considered with varying amounts of disorder. (**a**) The highly ordered and highly disordered categories; (**b**) the ordered and disordered categories; and (**c**) the percent disorder – where the disordered proteins (burgundy), the ordered proteins (mauve) and the proteome (yellow) are shown.

| 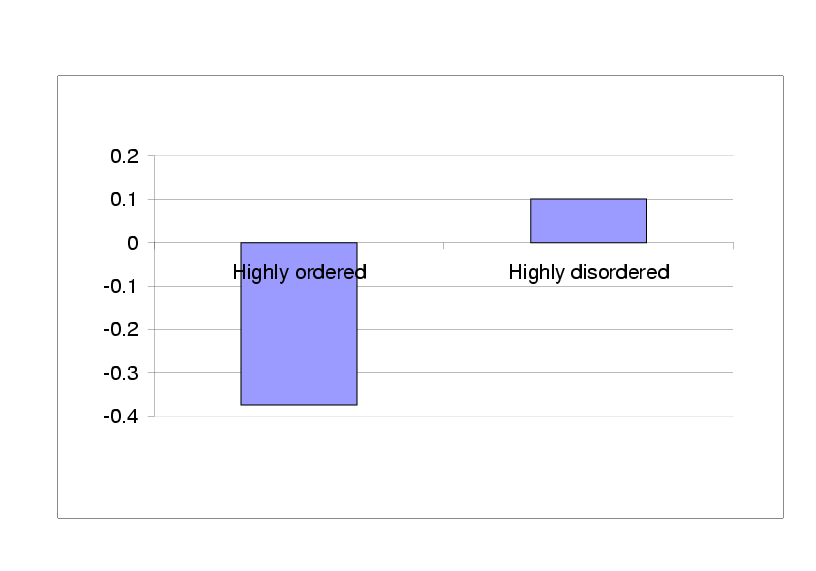 |
| --- |
| 1. See Figure 1c |
| 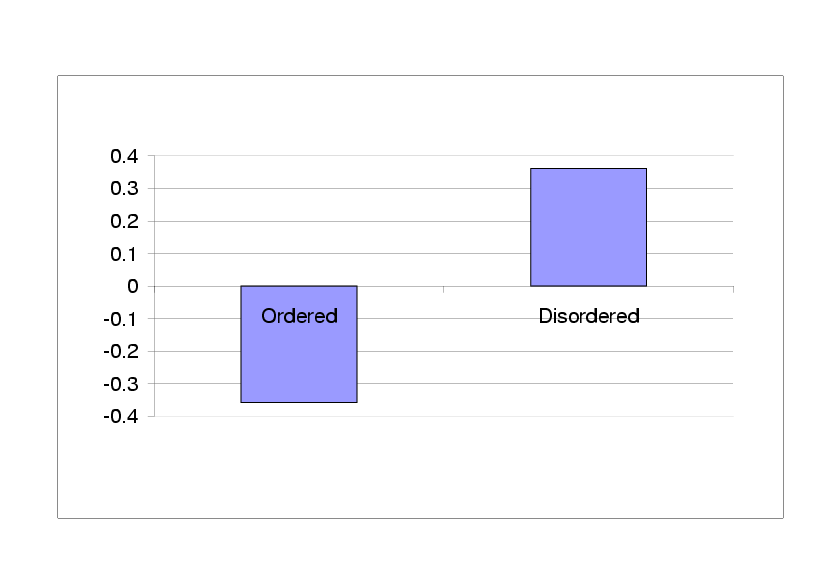 |
| 1. See Figure S1c |
| 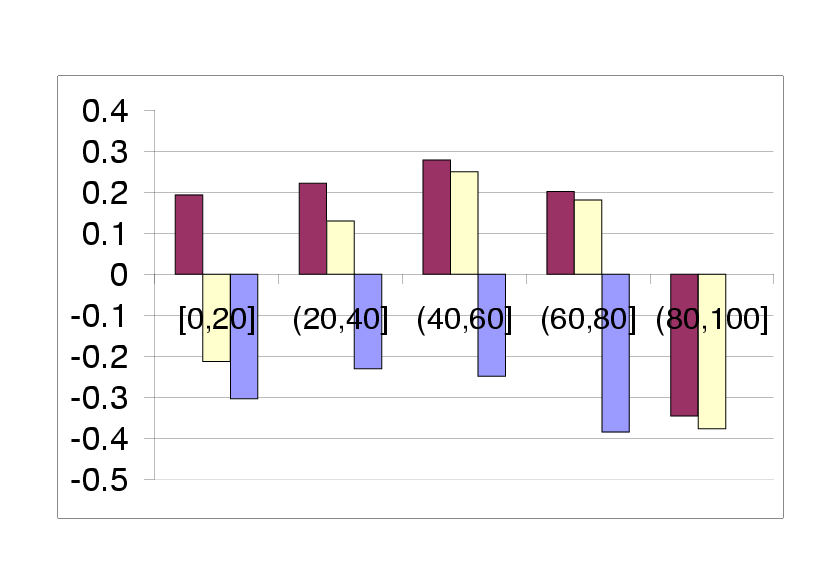 |
| (c) See Figure 2c |

**Figure S3. Biases in ubiquitination prediction with increase in protein disorder**. For proteins that have one or more ubiquitination sites, the logs odd-ratio (y-axis) versus the different datasets (x-axis) comprise varying amounts of disorder to highlight the disorder categories that are under represented and over represented. See key to **Figure S2**.

| 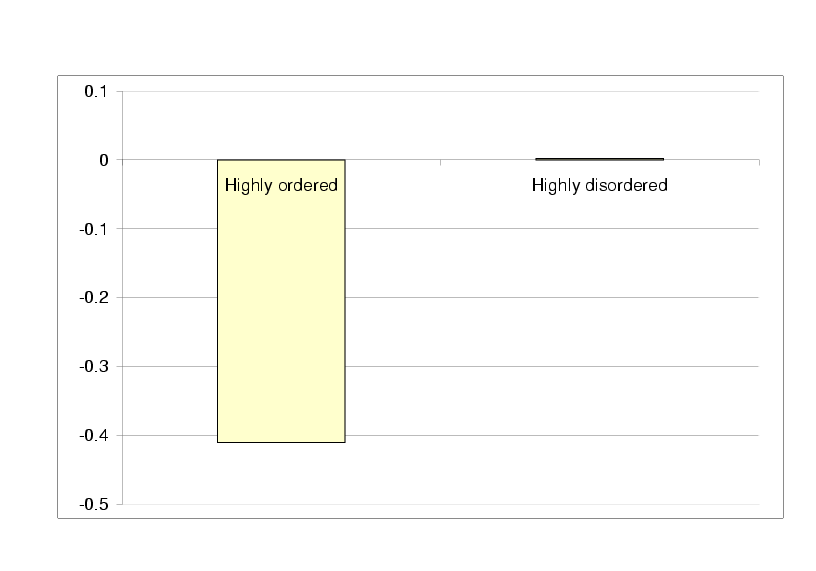 |
| --- |
| (a) See Figure 1d (bar chart) |
| 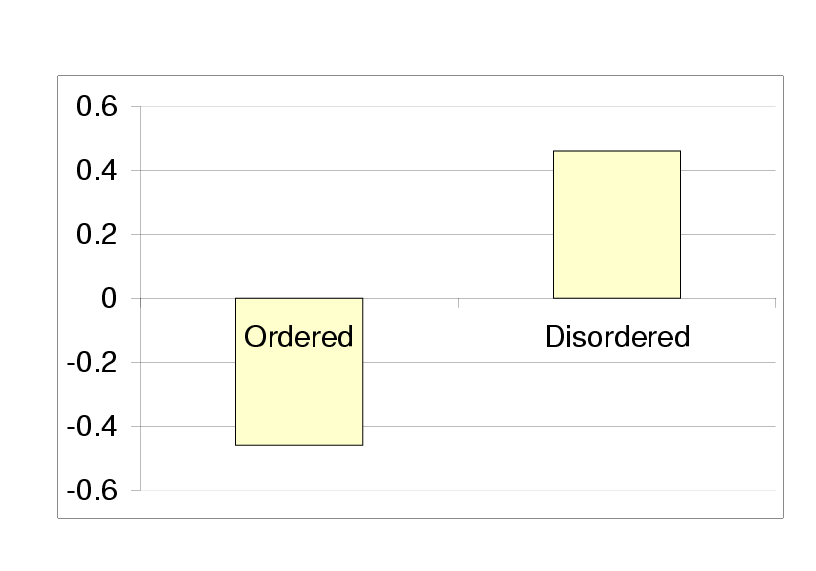 |
| (b) See Figure S1d (bar chart) |
| 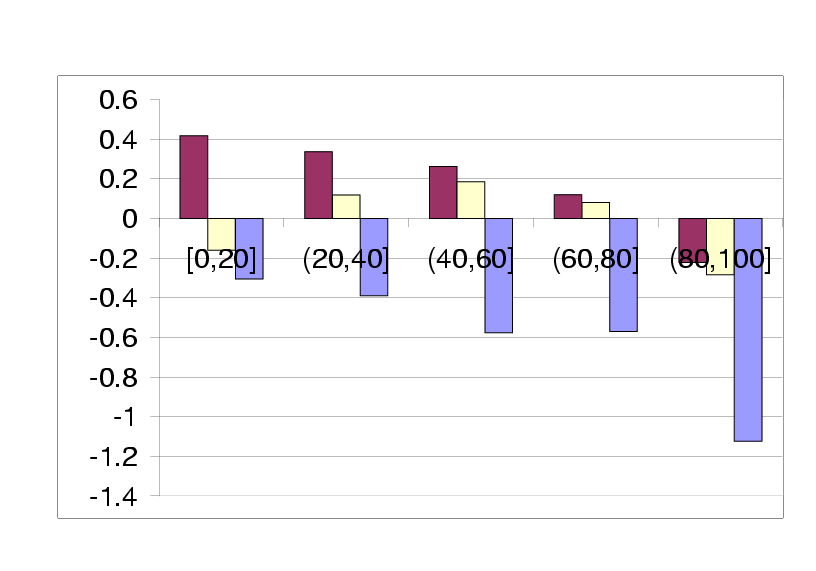 |
| (c) See Figure 2d (bar chart) |

**Figure S4.** **Bias in ubiquitination prediction with increase in protein disorder**. For the percent of predicted ubiquitination residues, the logs odd-ratio (y-axis) against the different datasets (x-axis) with varying amounts of disorder to highlight the disorder categories that are under represented and over represented in terms of ubiquitination. See key to **Figure S2**.

| 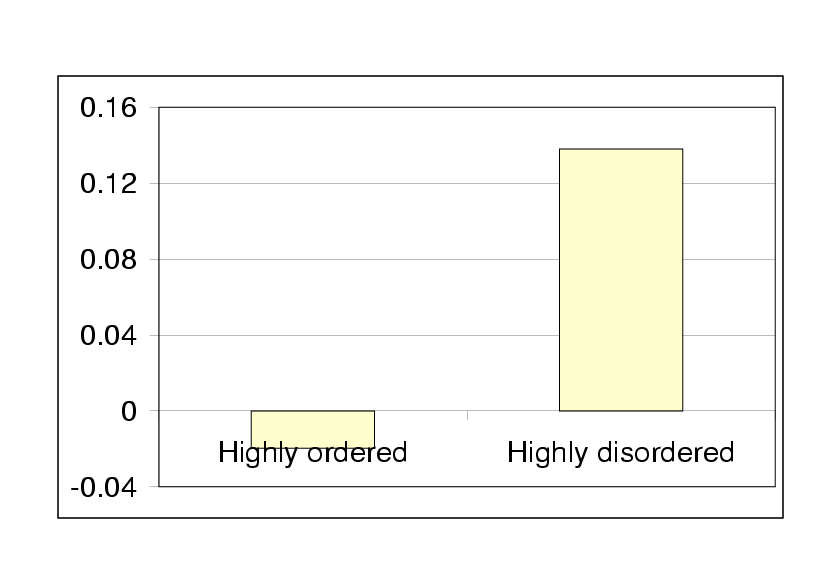 |
| --- |
| (a) See Figure 1d (line plot) |
| 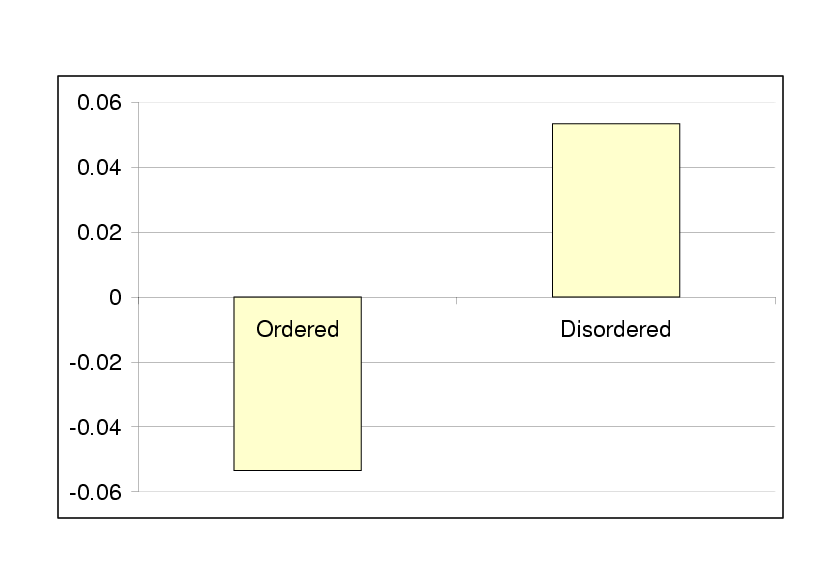 |
| (b) See Figure S1d (line plot) |
| 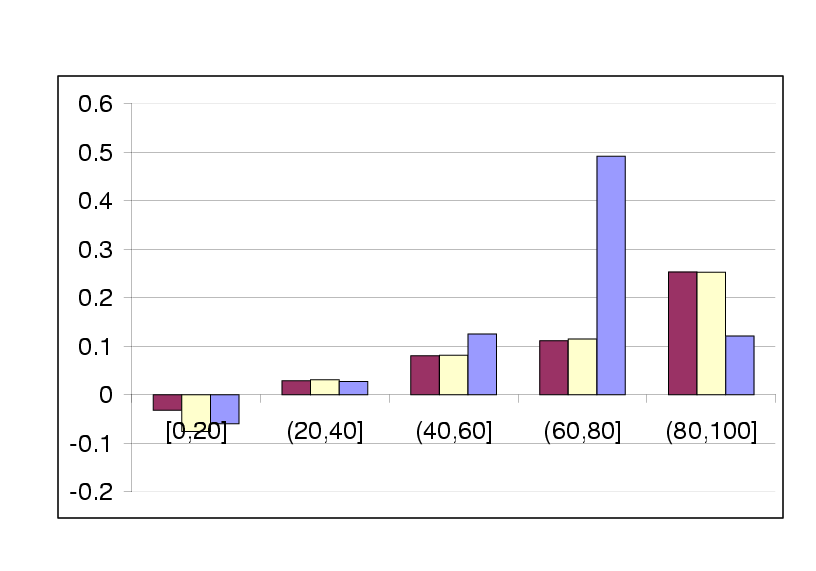 |
| (c) See Figure 2d (line plot) |

**Figure S5**. **The predicted ubiquitination sites and lysine composition with increase in protein disorder.** (**a**) The percent predicted ubiquitination sites (pink; primary axis) and the percent lysine composition (navy; secondary axis) in the five disordered categories. (**b**) The predicted ubiquitination sites normalised relative to the lysine frequency versus protein disorder.

| 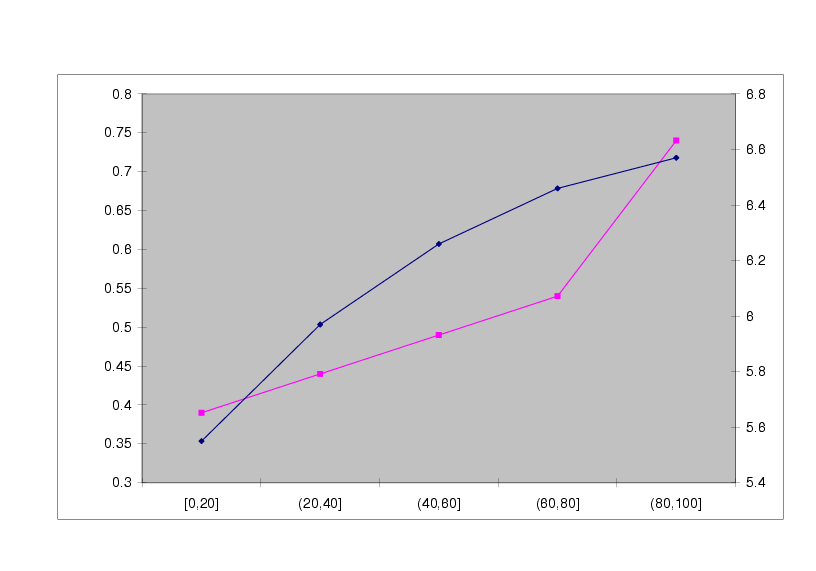 |
| --- |
| (a) |
| 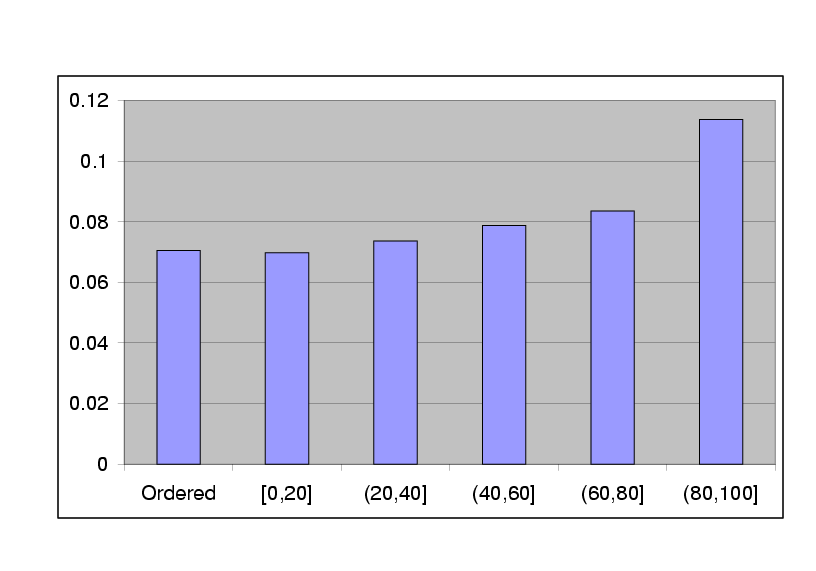 |
| (b) |

**Figure S6.** **Significance distribution and the critical values for correlation coefficients.** The Pearson correlation distribution for all the transcripts; the red line represents the mean of the correlation values and light red lines indicate the 5%, 1% and 0.1% critical regions of the distribution. The critical values of the correlation co-efficient are shown at the top of the graph in grey.


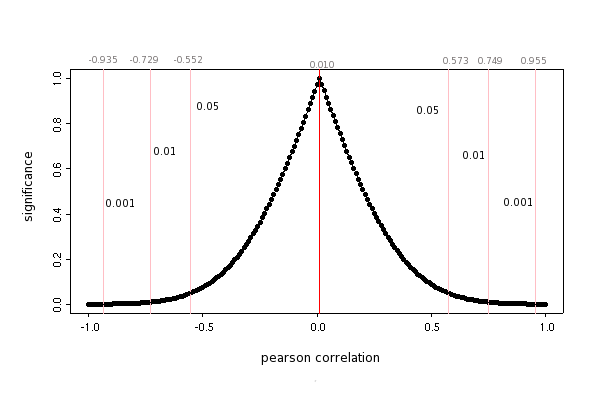


**Figure S7.** **Receiver operating characteristic (ROC) curve for the new ubiquitination predictor.** The x-axis represents the true positive rate and the y-axis represents the false positive rate.


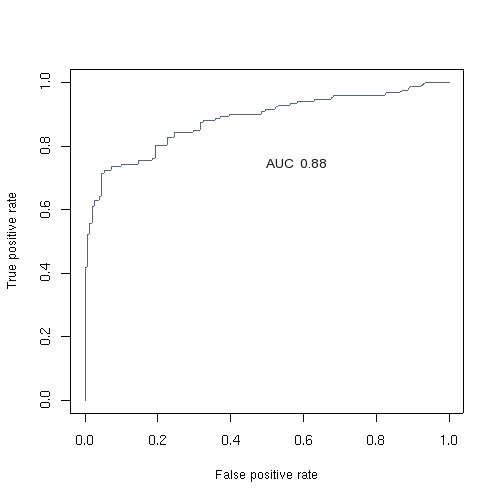

Supplement: Additional data file 1 — Figure S1 is a four part figure detailing the distributions of four properties, expression abundance, decay rate, and frequency of miRNA and ubiquitin target sites between disordered and ordered sequences. The plots are similar to those shown in Figure 1 but use alternative definitions of disorder and order to partition the data. Figure S2 is a plot of the occurrence of miRNA target sites as the amount of disorder increases. Figure S2a represents the occurrence of miRNA target sites in highly ordered and highly disordered sequences. Figure S2b represents the occurrence of miRNA target sites between ordered and disordered sequences and Figure S2c shows the occurrence of miRNA target sites as the amount of disorder increases. Figure S3 is a series of plots showing the frequency of sequences that are predicted to contain at least one ubiquitin target site. Figure S3a compares these frequencies between highly ordered and highly disordered sequences. Figure S3b is a similar plot between ordered and disordered sequences. Figure S3c is a bar plot of the frequency of ubiquitinated sequences in populations of disordered, ordered and all sequences as the amount of disorder increases. Figure S4 is a series of plots showing the frequency of predicted ubiquitin target sites in relation to varying amounts of disorder. Figure S4a is a bar plot of the frequency of ubiquitinated residues in highly disordered and highly ordered sequences. Figure S4b is a bar plot of the occurrence of ubiquitinated residues between ordered and disordered sequences. Figure S4c is a plot of the frequency of ubiquitinated residues in ordered, disordered and all sequences as the proportion of disordered residues increases. Figure S5a, b provides evidence that the predictions of ubiquitin target sites are independent of the proportion of lysine residues in the sequence despite the fact that both increase with the amount of disorder in the sequence. Figure S5a is a plot of the relationship between predicted [file gb-2009-10-5-r50-S1.doc]
