# Supplementary material for: Insights into the regulation of intrinsically disordered proteins in the human proteome by analyzing sequence and gene expression data
Source: Genome Biol. 2009 May 11;10(5):R50. doi: 10.1186/gb-2009-10-5-r50 (PMC2718516; doi:10.1186/gb-2009-10-5-r50)
Supplement: Additional data file 2 — Table S1 is a color coded listing of molecular function GO terms that are over-represented in clusters of disordered transcripts. Table S2 is a color coded listing of biological process GO terms that are over-represented in clusters of disordered transcripts. Tables S3a details the datasets used in the study and their composition when filtered for redundancy at 90% sequence identity. Table S3b lists the number of sequences that are classed as ordered and disordered when binned according to the amount of disorder present. Table S4 lists the ubiquitin target site predictions for the experimentally determined ligase target dataset. [file gb-2009-10-5-r50-S2.doc]

**Table S1**. Molecular Function GO terms over-represented in each transcript cluster encoding highly disordered proteins.

| **Group** | **GO term** | **Description** | **Odds** | **Pvalue** |
| --- | --- | --- | --- | --- |
|  | **GO:0046965** | retinoid X receptor binding | 567.35 | 3.78E-04 |
|  | **GO:0004862** | cAMP-dependent protein kinase inhibitor | Inf | 2.43E-02 |
|  | **GO:0051427** | Hormone receptor binding | 15.87 | 4.73E-02 |
|  | **GO:0042974** | retinoic acid receptor binding | 567.35 | 3.78E-04 |
|  | **GO:0008134** | transcription factor binding | 5.61 | 2.26E-02 |
|  | **GO:0030528** | transcription regulator | 7.08 | 3.59E-06 |
|  | **GO:0005198** | structural molecule | 3.76 | 3.24E-02 |
|  | **GO:0042975** | peroxisome proliferator activated receptor binding | 567.35 | 3.78E-04 |
|  | **GO:0003714** | transcription corepressor | 11.88 | 2.09E-02 |
|  | **GO:0043565** | sequence-specific DNA binding | 12.37 | 7.09E-07 |
|  | **GO:0030374** | ligand-dependent nuclear receptor coactivator | 51.90 | 8.12E-03 |
|  | **GO:0003677** | DNA binding | 5.33 | 3.52E-05 |
|  | **GO:0003700** | transcription factor | 8.70 | 2.25E-06 |
|  | **GO:0003680** | AT DNA binding | Inf | 2.31E-04 |
|  | **GO:0005201** | extracellular matrix structural constituent | 31.62 | 3.59E-06 |
|  | **GO:0035257** | nuclear hormone receptor binding | 16.33 | 4.73E-02 |
|  | **GO:0003712** | transcription cofactor | 7.46 | 8.12E-03 |
|  | **GO:0008147** | structural constituent of bone | 567.35 | 3.78E-04 |
|  | **GO:0004869** | Cysteine protease inhibitor | 117.31 | 6.08E-09 |
|  | **GO:0004857** | enzyme inhibitor | 13.02 | 2.46E-05 |
|  | **GO:0030234** | enzyme regulator | 4.99 | 4.11E-03 |
|  | **GO:0030161** | calpain inhibitor | Inf | 2.34E-13 |
|  | **GO:0004866** | endopeptidase inhibitor | 18.26 | 5.05E-05 |
|  | **GO:0030414** | Protease inhibitor | 18.08 | 5.05E-05 |
|  | **GO:0003891** | Delta DNA polymerase | 191.49 | 4.51E-02 |
|  | **GO:0043565** | sequence-specific DNA binding | 15.09 | 1.31E-06 |
|  | **GO:0003677** | DNA binding | 6.05 | 1.06E-04 |
|  | **GO:0003700** | transcription factor | 9.78 | 7.63E-06 |
|  | **GO:0016564** | transcriptional repressor | 8.48 | 4.51E-02 |
|  | **GO:0005201** | extracellular matrix structural constituent | 19.49 | 6.07E-03 |
|  | **GO:0030528** | transcription regulator | 8.61 | 7.63E-06 |
|  | **GO:0003735** | structural constituent of ribosome | Inf | 1.58E-02 |
|  | **GO:0005198** | structural molecule | Inf | 1.58E-02 |
|  | **GO:0003676** | nucleic acid binding | Inf | 8.03E-04 |
|  | **GO:0003723** | RNA binding | Inf | 1.01E-04 |
|  | **GO:0005488** | Binding | Inf | 1.58E-02 |
|  | **GO:0003682** | chromatin binding | 8.84 | 1.11E-03 |
|  | **GO:0030528** | transcription regulator | 12.18 | 2.64E-33 |
|  | **GO:0031490** | chromatin DNA binding | 172.06 | 4.77E-02 |
|  | **GO:0017049** | GTP-Rho binding | Inf | 1.61E-02 |
|  | **GO:0031267** | Small GTPase binding | 17.53 | 6.79E-05 |
|  | **GO:0003677** | DNA binding | 8.98 | 1.71E-27 |
|  | **GO:0003700** | transcription factor | 14.46 | 3.06E-34 |
|  | **GO:0017016** | Ras GTPase binding | 19.13 | 4.07E-05 |
|  | **GO:0017137** | Rab GTPase binding | 35.17 | 1.92E-03 |
|  | **GO:0043565** | sequence-specific DNA binding | 19.74 | 1.39E-35 |
|  | **GO:0051020** | GTPase binding | 15.78 | 1.25E-04 |
|  | **GO:0005201** | extracellular matrix structural constituent | 12.32 | 1.19E-04 |

**Table S2.** Biological Process GO terms enriched in transcript clusters encoding highly disordered proteins.

| **Group** | **GO term** | **Description** | **Odds** | **Pvalue** |
| --- | --- | --- | --- | --- |
|  | **GO:0001547** | antral ovarian follicle growth | Inf | 3.09E-02 |
|  | **GO:0019219** | regulation of nucleobase, side, tide and nucleic acid metabolic process | 4.10 | 5.81E-04 |
|  | **GO:0051246** | regulation of protein metabolic process | 12.53 | 2.61E-05 |
|  | **GO:0032984** | macromolecular complex disassembly | 56.55 | 8.18E-03 |
|  | **GO:0051129** | negative regulation of cell organization and biogenesis | 89.13 | 1.46E-09 |
|  | **GO:0031110** | regulation of microtubule polymerization or depolymerisation | 142.10 | 2.86E-09 |
|  | **GO:0048523** | negative regulation of cellular process | 4.32 | 4.52E-03 |
|  | **GO:0006337** | nucleosome disassembly | 251.04 | 1.14E-03 |
|  | **GO:0006820** | anion transport | 11.85 | 4.41E-04 |
|  | **GO:0045815** | positive regulation of gene expression, epigenetic | 251.04 | 1.14E-03 |
|  | **GO:0030837** | negative regulation of actin filament polymerization | Inf | 3.09E-02 |
|  | **GO:0006817** | phosphate transport | 37.98 | 1.39E-06 |
|  | **GO:0032956** | regulation of actin cytoskeleton organization and biogenesis | 18.21 | 4.30E-02 |
|  | **GO:0043624** | cellular protein complex disassembly | 56.55 | 8.18E-03 |
|  | **GO:0045449** | regulation of transcription | 4.25 | 4.35E-04 |
|  | **GO:0051128** | regulation of cellular component organization and biogenesis | 48.49 | 2.86E-09 |
|  | **GO:0008064** | regulation of actin polymerization and/or depolymerisation | 18.21 | 4.30E-02 |
|  | **GO:0022411** | cellular component disassembly | 21.24 | 3.67E-02 |
|  | **GO:0032508** | DNA duplex unwinding | 33.99 | 1.81E-02 |
|  | **GO:0006338** | chromatin remodelling | 18.21 | 4.30E-02 |
|  | **GO:0032392** | DNA geometric change | 33.99 | 1.81E-02 |
|  | **GO:0006355** | regulation of transcription, DNA-dependent | 4.60 | 1.86E-04 |
|  | **GO:0015698** | inorganic anion transport | 15.32 | 1.33E-04 |
|  | **GO:0051248** | negative regulation of protein metabolic process | 41.73 | 7.17E-09 |
|  | **GO:0032535** | regulation of cellular component size | 18.21 | 4.30E-02 |
|  | **GO:0031111** | negative regulation of microtubule de/polymerization | 212.50 | 8.66E-10 |
|  | **GO:0048519** | negative regulation of biological process | 4.60 | 1.71E-03 |
|  | **GO:0001544** | initiation of primordial ovarian follicle growth | Inf | 3.09E-02 |
|  | **GO:0042989** | sequestering of actin monomers | Inf | 3.09E-02 |
|  | **GO:0030832** | regulation of actin filament length | 18.21 | 4.30E-02 |
|  | **GO:0031498** | chromatin disassembly | 251.04 | 1.14E-03 |
|  | **GO:0009892** | negative regulation of metabolic process | 12.89 | 4.40E-07 |
|  | **GO:0031114** | regulation of microtubule depolymerisation | 212.50 | 8.66E-10 |
|  | **GO:0006268** | DNA unwinding during replication | 33.99 | 1.81E-02 |
|  | **GO:0006345** | loss of chromatin silencing | 502.25 | 6.84E-04 |
|  | **GO:0043241** | protein complex disassembly | 56.55 | 8.18E-03 |
|  | **GO:0007026** | negative regulation of microtubule depolymerisation | 212.50 | 8.66E-10 |
|  | **GO:0006397** | mRNA processing | 11.38 | 1.11E-02 |
|  | **GO:0033043** | regulation of organelle organization and biogenesis | 24.58 | 2.51E-02 |
|  | **GO:0008064** | regulation of actin polymerization and/or depolymerisation | 28.09 | 2.22E-02 |
|  | **GO:0006333** | chromatin assembly or disassembly | 10.59 | 1.11E-02 |
|  | **GO:0051016** | barbed-end actin filament capping | 65.44 | 1.11E-02 |
|  | **GO:0030834** | regulation of actin filament depolymerisation | 56.14 | 1.11E-02 |
|  | **GO:0006325** | establishment and/or maintenance of chromatin architecture | 8.72 | 1.11E-02 |
|  | **GO:0031497** | chromatin assembly | 10.26 | 2.72E-02 |
|  | **GO:0006323** | DNA packaging | 8.58 | 1.11E-02 |
|  | **GO:0006334** | nucleosome assembly | 11.11 | 2.48E-02 |
|  | **GO:0051017** | actin filament bundle formation | 98.07 | 1.11E-02 |
|  | **GO:0016071** | mRNA metabolic process | 9.72 | 1.32E-02 |
|  | **GO:0051493** | regulation of cytoskeleton organization and biogenesis | 24.58 | 2.51E-02 |
|  | **GO:0065004** | protein-DNA complex assembly | 7.95 | 5.00E-02 |
|  | **GO:0007001** | chromosome organization and biogenesis | 7.80 | 1.11E-02 |
|  | **GO:0051129** | negative regulation of cell organization and biogenesis | 28.09 | 2.22E-02 |
|  | **GO:0032535** | regulation of cellular component size | 28.09 | 2.22E-02 |
|  | **GO:0051276** | chromosome organization and biogenesis | 7.31 | 1.32E-02 |
|  | **GO:0008380** | RNA splicing | 14.42 | 1.11E-02 |
|  | **GO:0006996** | organelle organization and biogenesis | 5.65 | 1.11E-02 |
|  | **GO:0006396** | RNA processing | 8.06 | 1.11E-02 |
|  | **GO:0030832** | regulation of actin filament length | 28.09 | 2.22E-02 |
|  | **GO:0042255** | ribosome assembly | 187.22 | 4.97E-02 |
|  | **GO:0032956** | regulation of actin cytoskeleton organization and biogenesis | 28.09 | 2.22E-02 |
|  | **GO:0030835** | -ve regulation of actin filament depolymerisation | 60.43 | 1.11E-02 |
|  | **GO:0051693** | actin filament capping | 65.44 | 1.11E-02 |
|  | **GO:0051128** | regulation of cellular component organization and biogenesis | 22.32 | 1.11E-02 |
|  | **GO:0007015** | actin filament organization | 39.32 | 1.55E-02 |
|  | **GO:0045941** | positive regulation of transcription | 8.45 | 2.11E-02 |
|  | **GO:0045935** | +ve regulation of nucleobase side tide and nucleic acid metabolism | 8.25 | 2.11E-02 |
|  | **GO:0040001** | establishment of mitotic spindle localization | 194.28 | 3.80E-02 |
|  | **GO:0055007** | cardiac muscle cell differentiation | 194.28 | 3.80E-02 |
|  | **GO:0007275** | Multicellular organismal development | 9.75 | 2.50E-04 |
|  | **GO:0009887** | organ morphogenesis | 7.70 | 4.24E-02 |
|  | **GO:0009893** | positive regulation of metabolic process | 6.13 | 3.80E-02 |
|  | **GO:0006355** | regulation of transcription, DNA-dependent | 6.44 | 3.24E-04 |
|  | **GO:0015698** | inorganic anion transport | 11.20 | 2.61E-02 |
|  | **GO:0048732** | gland development | 32.82 | 2.11E-02 |
|  | **GO:0035051** | cardiac cell differentiation | 98.11 | 4.99E-02 |
|  | **GO:0030878** | thyroid gland development | 98.11 | 4.99E-02 |
|  | **GO:0045893** | +ve regulation of transcription, DNA-dependent | 7.38 | 4.47E-02 |
|  | **GO:0051293** | establishment of spindle localization | 130.44 | 4.47E-02 |
|  | **GO:0006820** | anion transport | 8.72 | 3.80E-02 |
|  | **GO:0035050** | embryonic heart tube development | 162.52 | 2.50E-03 |
|  | **GO:0035295** | tube development | 116.60 | 3.80E-03 |
|  | **GO:0000731** | DNA synthesis during DNA repair | 194.28 | 3.80E-02 |
|  | **GO:0010468** | regulation of gene expression | 6.48 | 2.80E-04 |
|  | **GO:0031325** | positive regulation of cellular metabolic process | 6.60 | 3.46E-02 |
|  | **GO:0048738** | cardiac muscle development | 98.11 | 4.99E-02 |
|  | **GO:0001947** | heart looping | 194.28 | 3.80E-02 |
|  | **GO:0032502** | developmental process | 3.65 | 2.11E-02 |
|  | **GO:0051146** | striated muscle cell differentiation | 98.11 | 4.99E-02 |
|  | **GO:0006817** | phosphate transport | 26.77 | 4.45E-03 |
|  | **GO:0031323** | regulation of cellular metabolic process | 6.10 | 3.42E-04 |
|  | **GO:0033205** | Cytokinesis during cell cycle | 384.43 | 3.46E-02 |
|  | **GO:0045449** | regulation of transcription | 6.99 | 2.50E-04 |
|  | **GO:0000281** | Cytokinesis after mitosis | 384.43 | 3.46E-02 |
|  | **GO:0009888** | tissue development | 8.43 | 3.80E-02 |
|  | **GO:0048856** | anatomical structure development | 4.47 | 2.28E-02 |
|  | **GO:0006357** | regulation of transcription from RNA pol II promoter | 5.67 | 3.89E-02 |
|  | **GO:0044260** | cellular macromolecule metabolic process | Inf | 2.43E-02 |
|  | **GO:0019538** | protein metabolic process | Inf | 2.45E-02 |
|  | **GO:0006412** | Translation | Inf | 2.19E-04 |
|  | **GO:0045941** | positive regulation of transcription | 4.14 | 6.27E-03 |
|  | **GO:0045935** | +ve regulation of nucleobase side tide and nucleic acid metabolism | 4.56 | 2.22E-03 |
|  | **GO:0017156** | calcium ion-dependent exocytosis | 19.85 | 4.73E-02 |
|  | **GO:0001654** | Eye development | 12.29 | 2.30E-02 |
|  | **GO:0048513** | organ development | 7.79 | 1.46E-11 |
|  | **GO:0007275** | Multicellular organismal development | 13.45 | 6.09E-27 |
|  | **GO:0031274** | positive regulation of pseudopodium formation | Inf | 1.84E-03 |
|  | **GO:0006351** | transcription, DNA-dependent | 7.81 | 1.89E-05 |
|  | **GO:0007423** | sensory organ development | 12.29 | 2.30E-02 |
|  | **GO:0045934** | -ve regulation of nucleobase side tide and nucleic acid metabolism | 4.56 | 2.22E-03 |
|  | **GO:0031272** | regulation of pseudopodium formation | Inf | 1.84E-03 |
|  | **GO:0032502** | developmental process | 6.32 | 3.90E-18 |
|  | **GO:0050793** | regulation of developmental process | 4.82 | 5.11E-03 |
|  | **GO:0048856** | anatomical structure development | 5.77 | 7.04E-12 |
|  | **GO:0006357** | regulation of transcription from RNA pol II promoter | 4.60 | 1.96E-04 |
|  | **GO:0001708** | Cell fate specification | 27.04 | 4.18E-03 |
|  | **GO:0003002** | regionalization | 6.26 | 3.86E-02 |
|  | **GO:0016481** | negative regulation of transcription | 4.43 | 4.38E-03 |
|  | **GO:0045892** | negative regulation of transcription, DNA-dependent | 5.55 | 2.63E-03 |
|  | **GO:0030198** | extracellular matrix organization and biogenesis | 10.81 | 2.83E-02 |
|  | **GO:0015698** | inorganic anion transport | 7.90 | 1.30E-04 |
|  | **GO:0035107** | Appendage morphogenesis | 16.40 | 5.51E-04 |
|  | **GO:0048858** | Cell projection morphogenesis | 11.76 | 2.46E-02 |
|  | **GO:0030326** | embryonic limb morphogenesis | 14.56 | 3.47E-03 |
|  | **GO:0035108** | Limb morphogenesis | 16.40 | 5.51E-04 |
|  | **GO:0007409** | axonogenesis | 14.24 | 1.66E-02 |
|  | **GO:0030154** | Cell differentiation | 4.60 | 1.89E-05 |
|  | **GO:0007517** | muscle development | 6.18 | 1.71E-02 |
|  | **GO:0001501** | skeletal development | 12.06 | 7.18E-06 |
|  | **GO:0006350** | transcription | 3.73 | 3.54E-06 |
|  | **GO:0048731** | system development | 3.89 | 3.50E-03 |
|  | **GO:0031344** | regulation of cell projection organization and biogenesis | 177.77 | 4.19E-03 |
|  | **GO:0032774** | RNA biosynthetic process | 7.69 | 2.06E-05 |
|  | **GO:0045944** | positive regulation of transcription from RNA pol II promoter | 6.32 | 2.89E-03 |
|  | **GO:0031346** | positive regulation of cell projection organization and biogenesis | Inf | 1.84E-03 |
|  | **GO:0001764** | neuron migration | 10.81 | 2.83E-02 |
|  | **GO:0009893** | positive regulation of metabolic process | 3.75 | 4.38E-03 |
|  | **GO:0022604** | regulation of cell morphogenesis | 10.81 | 2.83E-02 |
|  | **GO:0006366** | transcription from RNA polymerase II promoter | 7.78 | 5.19E-05 |
|  | **GO:0007420** | brain development | 15.89 | 1.26E-04 |
|  | **GO:0009653** | anatomical structure morphogenesis | 6.74 | 1.38E-09 |
|  | **GO:0007389** | pattern specification process | 8.00 | 3.52E-04 |
|  | **GO:0006820** | anion transport | 6.08 | 7.62E-04 |
|  | **GO:0007417** | central nervous system development | 9.91 | 1.10E-03 |
|  | **GO:0006139** | Nucleobase, side, tide and nucleic acid metabolic process | 2.25 | 1.84E-03 |
|  | **GO:0006817** | phosphate transport | 20.44 | 1.39E-07 |
|  | **GO:0030182** | neuron differentiation | 15.82 | 2.80E-03 |
|  | **GO:0000122** | negative regulation of transcription from RNA pol II promoter | 6.78 | 2.18E-03 |
|  | **GO:0016070** | RNA metabolic process | 2.41 | 4.29E-02 |
|  | **GO:0045449** | regulation of transcription | 10.77 | 4.48E-29 |
|  | **GO:0022603** | regulation of anatomical structure morphogenesis | 10.81 | 2.83E-02 |
|  | **GO:0009887** | organ morphogenesis | 5.34 | 1.81E-03 |
|  | **GO:0006355** | regulation of transcription, DNA-dependent | 10.76 | 4.48E-29 |
|  | **GO:0035113** | embryonic appendage morphogenesis | 14.56 | 3.47E-03 |
|  | **GO:0031324** | negative regulation of cellular metabolic process | 3.87 | 5.77E-03 |
|  | **GO:0045893** | positive regulation of transcription, DNA-dependent | 4.47 | 7.44E-03 |
|  | **GO:0048812** | neurite morphogenesis | 11.76 | 2.46E-02 |
|  | **GO:0031325** | positive regulation of cellular metabolic process | 3.63 | 8.43E-03 |
|  | **GO:0008360** | regulation of cell shape | 10.81 | 2.83E-02 |
|  | **GO:0009892** | negative regulation of metabolic process | 3.35 | 1.46E-02 |
|  | **GO:0042472** | inner ear morphogenesis | 38.27 | 2.19E-05 |
|  | **GO:0048598** | embryonic morphogenesis | 8.18 | 6.00E-03 |

**Table S3**. Percentage of transcripts encoding disordered proteins likely to be targeted by miRNA. For each dataset, the total number of transcripts encoding proteins^a^ and the number of unique protein sequences^b^ encoded by transcripts are given. A match^c^ occurs when a transcript of a protein sequence matches an mRNA targeted by a miRNA. The percentage^d^ calculations are described in the methods. **(a)** The types of disorder (**Figures 1d, 2d).** **(b)** The percentages of disordered residues (**Figure 3c)**.

**(a)**

| Data Set | Removal of proteins that share 90% or higher sequence identity  using CD Hit - Li & Godzik (2006), Bioinformatics 22:1658-1659. | | | |
| --- | --- | --- | --- | --- |
| Type of disorder | Total^a^ | Unique^b^ | Match^c^ | Percentage^d^ |
|  |  |  |  |  |
| Highly disordered | 667 | 667 | 197 | **29.54** |
| Highly ordered | 4363 | 4363 | 649 | **14.88** |
|  |  |  |  |  |
| Disordered | 11477 | 11477 | 4118 | **35.88** |
| Ordered | 14534 | 14534 | 2858 | **19.66** |
| All proteins | 26011 | 26011 | 6976 | **26.82** |
|  |  |  |  |  |
| Completely ordered | 485 | 485 | 45 | **9.28** |
| Completely  Disordered | 21 | 21 | 6 | **28.57** |

**(b)**

| Data Set | Removal of proteins that share 90% or higher sequence identity using CD Hit (Li & Godzik, 2006) | | | |
| --- | --- | --- | --- | --- |
| Percentage disorder | Total^a^ | Unique^b^ | Match^c^ | Percentage^d^ |
|  |  |  |  |  |
| **Disordered** |  |  |  |  |
| [0,20] | 3316 | 3316 | 1151 | **34.71** |
| [20,40] | 5276 | 5276 | 1883 | **35.69** |
| [40,60] | 2253 | 2253 | 890 | **39.50** |
| [60,80] | 522 | 522 | 181 | **34.67** |
| [80,100] | 110 | 110 | 13 | **11.82** |
| Total | 11477 | 11477 | 4118 | **35.88** |
|  |  |  |  |  |
| **Ordered** |  |  |  |  |
| [0,20] | 12664 | 12664 | 2534 | **20.01** |
| [20,40] | 1656 | 1656 | 291 | **17.57** |
| [40,60] | 179 | 179 | 30 | **16.76** |
| [60,80] | 26 | 26 | 3 | **11.54** |
| [80,100] | 9 | 9 | 0 | **0** |
| Total | 14534 | 14534 | 2858 | **19.66** |
|  |  |  |  |  |
| **Proteome** |  |  |  |  |
| [0,20] | 12664 | 1266 | 3682 | **23.05** |
| [20,40] | 6930 | 6930 | 2177 | **31.41** |
| [40,60] | 2440 | 2440 | 920 | **37.70** |
| [60,80] | 547 | 547 | 184 | **33.64** |
| [80,100] | 120 | 120 | 13 | **10.83** |
| Total | 26011 | 26011 | 6976 | **26.82** |

**Table S4**. **The extent of overlap of the proteins predicted to be ubiquinated and the identified targets of the SCF ubiquitin ligase**. The method and result for this investigation is described in the “Protein Disorder and miRNA targets” section.

| **GENE_ID** | **GENE_SYMBOL** | **MAPPED_ACCESSION** | **UBQ_RESIDUE** |
| --- | --- | --- | --- |
| 26751 | SH3YL1 | ENSP00000384910 | 34 |
| 84445 | LZTS2 | ENSP00000359243 | 526 |
| 9238 | TBRG4 | ENSP00000379016 | 230 |
| 9238 | TBRG4 | ENSP00000379016 | 399 |
| 9238 | TBRG4 | ENSP00000379016 | 498 |
| 3853 | KRT6A | ENSP00000369317 | 338 |
| 3853 | KRT6A | ENSP00000369317 | 436 |
| 55526 | DHTKD1 | ENSP00000263035 | 37 |
| 55526 | DHTKD1 | ENSP00000263035 | 72 |
| 55526 | DHTKD1 | ENSP00000263035 | 244 |
| 55526 | DHTKD1 | ENSP00000263035 | 881 |
| 9946 | CRYZL1 | ENSP00000370966 | 344 |
| 7289 | TULP3 | ENSP00000380321 | 35 |
| 7289 | TULP3 | ENSP00000380321 | 59 |
| 7289 | TULP3 | ENSP00000380321 | 92 |
| 7289 | TULP3 | ENSP00000380321 | 287 |
| 7289 | TULP3 | ENSP00000380321 | 436 |
| 10948 | STARD3 | ENSP00000377794 | 242 |
| 60680 | BRUNOL5 | ENSP00000335182 | 40 |
| 28231 | SLCO4A1 | ENSP00000359538 | 56 |
| 28231 | SLCO4A1 | ENSP00000359538 | 640 |
| 4793 | NFKBIB | ENSP00000375929 | 135 |
| 4793 | NFKBIB | ENSP00000375929 | 258 |
| 285172 | MGC39518 | ENSP00000286181 | 132 |
| 285172 | MGC39518 | ENSP00000286181 | 504 |
| 285172 | MGC39518 | ENSP00000286181 | 515 |
| 5690 | PSMB2 | ENSP00000362337 | 158 |
| 79873 | NUDT18 | ENSP00000307852 | 5 |
| 79873 | NUDT18 | ENSP00000307852 | 28 |
| 79873 | NUDT18 | ENSP00000307852 | 307 |
| 79873 | NUDT18 | ENSP00000307852 | 466 |
| 11083 | DATF1 | ENSP00000378749 | 4 |
| 11083 | DATF1 | ENSP00000378749 | 141 |
| 11083 | DATF1 | ENSP00000378749 | 247 |
| 11083 | DATF1 | ENSP00000378749 | 339 |
| 11083 | DATF1 | ENSP00000378749 | 372 |
| 11083 | DATF1 | ENSP00000378749 | 446 |
| 11083 | DATF1 | ENSP00000378749 | 515 |
| 11083 | DATF1 | ENSP00000378749 | 540 |
| 11083 | DATF1 | ENSP00000378749 | 1182 |
| 57125 | PLXDC1 | ENSP00000377853 | 47 |
| 27229 | 76P | ENSP00000382387 | 503 |
| 4722 | NDUFS3 | ENSP00000263774 | 56 |
| 4722 | NDUFS3 | ENSP00000263774 | 144 |
| 4722 | NDUFS3 | ENSP00000263774 | 259 |
| 54981 | C9orf95 | ENSP00000366004 | 40 |
| 54981 | C9orf95 | ENSP00000366004 | 49 |
| 5098 | PCDHGC3 | ENSP00000306918 | 156 |
| 5098 | PCDHGC3 | ENSP00000306918 | 648 |
| 5098 | PCDHGC3 | ENSP00000306918 | 920 |
| 27132 | CPNE7 | ENSP00000317374 | 133 |
| 27132 | CPNE7 | ENSP00000317374 | 521 |
| 4129 | MAOB | ENSP00000380428 | 21 |
| 4129 | MAOB | ENSP00000380428 | 230 |
| 126961 | HIST2H3C | ENSP00000358154 | 123 |
| 22977 | AKR7A3 | ENSP00000355377 | 136 |
| 22977 | AKR7A3 | ENSP00000355377 | 250 |
| 993 | CDC25A | ENSP00000343166 | 28 |
| 993 | CDC25A | ENSP00000343166 | 66 |
| 993 | CDC25A | ENSP00000343166 | 141 |
| 5725 | PTBP1 | ENSP00000349428 | 46 |
| 6500 | SKP1A | ENSP00000231487 | 5 |
| 6500 | SKP1A | ENSP00000231487 | 28 |
| 81614 | NIPA2 | ENSP00000381096 | 210 |
| 5283 | PIGH | ENSP00000216452 | 161 |
| 57628 | DPP10 | ENSP00000386565 | 137 |
| 57628 | DPP10 | ENSP00000386565 | 697 |
| 57628 | DPP10 | ENSP00000386565 | 715 |
| 9545 | RAB3D | ENSP00000222120 | 121 |
| 84735 | CNDP1 | ENSP00000351682 | 167 |
| 84735 | CNDP1 | ENSP00000351682 | 455 |
| 84735 | CNDP1 | ENSP00000351682 | 483 |
| 80020 | FLJ23322 | ENSP00000380401 | 681 |
| 54784 | FLJ20013 | ENSP00000292566 | 117 |
| 23172 | KIAA0157 | ENSP00000357827 | 10 |
| 2870 | GRK6 | ENSP00000377204 | 14 |
| 9653 | HS2ST1 | ENSP00000359582 | 126 |
| 9653 | HS2ST1 | ENSP00000359582 | 135 |
| 54956 | PARP16 | ENSP00000261888 | 80 |
| 54956 | PARP16 | ENSP00000261888 | 233 |
| 2132 | EXT2 | ENSP00000379032 | 6 |
| 2132 | EXT2 | ENSP00000379032 | 285 |
| 51307 | FAM53C | ENSP00000239906 | 304 |
| 50613 | UBQLN3 | ENSP00000347997 | 32 |
| 54935 | DUSP23 | ENSP00000357089 | 140 |
| 54518 | APBB1IP | ENSP00000365411 | 87 |
| 54518 | APBB1IP | ENSP00000365411 | 254 |
| 54518 | APBB1IP | ENSP00000365411 | 289 |
| 54518 | APBB1IP | ENSP00000365411 | 373 |
| 54518 | APBB1IP | ENSP00000365411 | 421 |
| 54518 | APBB1IP | ENSP00000365411 | 493 |
| 84869 | CBR4 | ENSP00000303525 | 196 |
| 54470 | ARMCX6 | ENSP00000378560 | 101 |
| 54470 | ARMCX6 | ENSP00000378560 | 257 |
| 1819 | DRG2 | ENSP00000379076 | 46 |
| 1819 | DRG2 | ENSP00000379076 | 172 |
| 1026 | CDKN1A | ENSP00000384849 | 161 |
| 9411 | ARHGAP29 | ENSP00000359237 | 366 |
| 9411 | ARHGAP29 | ENSP00000359237 | 371 |
| 79083 | MLPH | ENSP00000386338 | 3 |
| 79083 | MLPH | ENSP00000386338 | 44 |
| 79083 | MLPH | ENSP00000386338 | 300 |
| 79083 | MLPH | ENSP00000386338 | 441 |
| 122011 | CSNK1A1L | ENSP00000369126 | 46 |
| 122011 | CSNK1A1L | ENSP00000369126 | 325 |
| 7517 | XRCC3 | ENSP00000343392 | 18 |
| 115704 | EVI5L | ENSP00000270530 | 109 |
| 11135 | CDC42EP1 | ENSP00000249014 | 390 |
| 8772 | FADD | ENSP00000301838 | 153 |
| 27250 | PDCD4 | ENSP00000376816 | 53 |
| 27250 | PDCD4 | ENSP00000376816 | 84 |
| 27250 | PDCD4 | ENSP00000376816 | 103 |
| 27250 | PDCD4 | ENSP00000376816 | 233 |
| 27250 | PDCD4 | ENSP00000376816 | 324 |
| 27250 | PDCD4 | ENSP00000376816 | 453 |
| 7287 | TULP1 | ENSP00000362999 | 247 |
| 7287 | TULP1 | ENSP00000362999 | 331 |
| 7287 | TULP1 | ENSP00000362999 | 536 |
| 7867 | MAPKAPK3 | ENSP00000350639 | 36 |
| 7867 | MAPKAPK3 | ENSP00000350639 | 192 |
| 7867 | MAPKAPK3 | ENSP00000350639 | 332 |
| 10487 | CAP1 | ENSP00000383844 | 149 |
| 30833 | NT5C | ENSP00000245552 | 194 |
| 3728 | JUP | ENSP00000377508 | 124 |
| 3728 | JUP | ENSP00000377508 | 303 |
| 64093 | SMOC1 | ENSP00000370680 | 165 |
| 64093 | SMOC1 | ENSP00000370680 | 223 |
| 64093 | SMOC1 | ENSP00000370680 | 414 |
| 56000 | NXF3 | ENSP00000378504 | 199 |
| 148022 | TICAM1 | ENSP00000344347 | 3 |
| 148022 | TICAM1 | ENSP00000344347 | 538 |
| 54762 | DKFZp434C0328 | ENSP00000350881 | 300 |
| 27242 | TNFRSF21 | ENSP00000296861 | 342 |
| 27242 | TNFRSF21 | ENSP00000296861 | 420 |
| 27242 | TNFRSF21 | ENSP00000296861 | 529 |
| 11267 | SNF8 | ENSP00000290330 | 13 |
| 54984 | PINX1 | ENSP00000346908 | 39 |
| 54984 | PINX1 | ENSP00000346908 | 59 |
| 9725 | TMEM63A | ENSP00000355800 | 378 |
| 9725 | TMEM63A | ENSP00000355800 | 598 |
| 9725 | TMEM63A | ENSP00000355800 | 658 |
| 9725 | TMEM63A | ENSP00000355800 | 729 |
| 27044 | SND1 | ENSP00000346762 | 25 |
| 27044 | SND1 | ENSP00000346762 | 310 |
| 27044 | SND1 | ENSP00000346762 | 359 |
| 27044 | SND1 | ENSP00000346762 | 488 |
| 27044 | SND1 | ENSP00000346762 | 603 |
| 27044 | SND1 | ENSP00000346762 | 752 |
| 10208 | C13orf22 | ENSP00000255304 | 516 |
| 10208 | C13orf22 | ENSP00000255304 | 709 |
| 1831 | TSC22D3 | ENSP00000378386 | 51 |
| 79912 | PYROXD1 | ENSP00000240651 | 148 |
| 79912 | PYROXD1 | ENSP00000240651 | 201 |
| 79912 | PYROXD1 | ENSP00000240651 | 448 |
| 10949 | HNRPA0 | ENSP00000316042 | 133 |
| 10949 | HNRPA0 | ENSP00000316042 | 171 |
| 10949 | HNRPA0 | ENSP00000316042 | 269 |
| 51225 | ABI3 | ENSP00000225941 | 169 |
| 25804 | LSM4 | ENSP00000252816 | 138 |
| 80149 | ZC3H12A | ENSP00000362179 | 204 |
| 80149 | ZC3H12A | ENSP00000362179 | 273 |
| 80149 | ZC3H12A | ENSP00000362179 | 551 |
| 2257 | FGF12 | ENSP00000376248 | 174 |
| 84263 | HSDL2 | ENSP00000381783 | 29 |
| 84263 | HSDL2 | ENSP00000381783 | 32 |
| 84263 | HSDL2 | ENSP00000381783 | 42 |
| 84263 | HSDL2 | ENSP00000381783 | 99 |
| 84263 | HSDL2 | ENSP00000381783 | 245 |
| 23212 | RRS1 | ENSP00000322396 | 12 |
| 23212 | RRS1 | ENSP00000322396 | 127 |
| 23212 | RRS1 | ENSP00000322396 | 178 |
| 23212 | RRS1 | ENSP00000322396 | 198 |
| 23212 | RRS1 | ENSP00000322396 | 279 |
| 23212 | RRS1 | ENSP00000322396 | 362 |
| 92715 | C9orf112 | ENSP00000277540 | 57 |
| 440 | ASNS | ENSP00000377845 | 191 |
| 56675 | NRIP3 | ENSP00000379879 | 30 |
| 56675 | NRIP3 | ENSP00000379879 | 38 |
| 56675 | NRIP3 | ENSP00000379879 | 192 |
| 56675 | NRIP3 | ENSP00000379879 | 237 |
| 57687 | KIAA1576 | ENSP00000303129 | 217 |
| 84948 | TIGD5 | ENSP00000315906 | 5 |
| 84948 | TIGD5 | ENSP00000315906 | 44 |
| 84948 | TIGD5 | ENSP00000315906 | 155 |
| 84948 | TIGD5 | ENSP00000315906 | 261 |
| 219654 | C10orf56 | ENSP00000361410 | 267 |
| 808 | CALM3 | ENSP00000375785 | 22 |
| 808 | CALM3 | ENSP00000375785 | 76 |
| 808 | CALM3 | ENSP00000375785 | 95 |
| 284058 | LOC284058 | ENSP00000377117 | 93 |
| 284058 | LOC284058 | ENSP00000377117 | 262 |
| 5307 | PITX1 | ENSP00000265340 | 143 |
| 84958 | SYTL1 | ENSP00000363171 | 41 |
| 1937 | EEF1G | NP_001395.1 | 275 |
| 1937 | EEF1G | NP_001395.1 | 354 |
| 222171 | LOC222171 | ENSP00000317836 | 128 |
| 148932 | MOBKL2C | ENSP00000379586 | 6 |
| 23117 | LOC23117 | NP_569731.1 | 673 |
| 7171 | TPM4 | ENSP00000384985 | 11 |
| 7171 | TPM4 | ENSP00000384985 | 132 |
| 7171 | TPM4 | ENSP00000384985 | 153 |
| 7171 | TPM4 | ENSP00000384985 | 215 |
| 53340 | SPA17 | ENSP00000227135 | 95 |
| 53340 | SPA17 | ENSP00000227135 | 111 |
| 53340 | SPA17 | ENSP00000227135 | 120 |
| 53340 | SPA17 | ENSP00000227135 | 138 |
| 5157 | PDGFRL | ENSP00000381149 | 2 |
| 25945 | PVRL3 | ENSP00000321514 | 77 |
| 25945 | PVRL3 | ENSP00000321514 | 290 |
| 25945 | PVRL3 | ENSP00000321514 | 471 |
| 25945 | PVRL3 | ENSP00000321514 | 519 |
| 4723 | NDUFV1 | ENSP00000322450 | 36 |
| 89822 | KCNK17 | ENSP00000362328 | 175 |
| 89822 | KCNK17 | ENSP00000362328 | 330 |
| 55276 | PGM2 | ENSP00000371393 | 333 |
| 55276 | PGM2 | ENSP00000371393 | 360 |
| 55276 | PGM2 | ENSP00000371393 | 410 |
| 6838 | SURF6 | ENSP00000361092 | 87 |
| 6838 | SURF6 | ENSP00000361092 | 260 |
| 11054 | OGFR | ENSP00000349686 | 283 |
| 11054 | OGFR | ENSP00000349686 | 384 |
| 1016 | CDH18 | ENSP00000371710 | 89 |
| 1016 | CDH18 | ENSP00000371710 | 158 |
| 1016 | CDH18 | ENSP00000371710 | 371 |
| 1016 | CDH18 | ENSP00000371710 | 640 |
| 9915 | ARNT2 | ENSP00000378190 | 57 |
| 9915 | ARNT2 | ENSP00000378190 | 119 |
| 9915 | ARNT2 | ENSP00000378190 | 271 |
| 9915 | ARNT2 | ENSP00000378190 | 276 |
| 9915 | ARNT2 | ENSP00000378190 | 565 |
| 59338 | PLEKHA1 | ENSP00000386800 | 350 |
| 5970 | RELA | ENSP00000384273 | 452 |
| 8572 | PDLIM4 | ENSP00000368303 | 206 |
| 5064 | PALM | ENSP00000379262 | 4 |
| 5064 | PALM | ENSP00000379262 | 175 |
| 5064 | PALM | ENSP00000379262 | 193 |
| 5064 | PALM | ENSP00000379262 | 360 |
| 10067 | SCAMP3 | ENSP00000347540 | 287 |
| 51187 | C15orf15 | ENSP00000260443 | 5 |
| 51187 | C15orf15 | ENSP00000260443 | 57 |
| 51187 | C15orf15 | ENSP00000260443 | 73 |
| 51187 | C15orf15 | ENSP00000260443 | 94 |
| 8509 | NDST2 | ENSP00000299641 | 588 |
| 8509 | NDST2 | ENSP00000299641 | 697 |
| 8509 | NDST2 | ENSP00000299641 | 813 |
| 84219 | WDR24 | ENSP00000293883 | 258 |
| 84219 | WDR24 | ENSP00000293883 | 771 |
| 85007 | MGC15875 | ENSP00000377128 | 125 |
| 85007 | MGC15875 | ENSP00000377128 | 187 |
| 85007 | MGC15875 | ENSP00000377128 | 297 |
| 166614 | DCAMKL2 | ENSP00000377578 | 40 |
| 166614 | DCAMKL2 | ENSP00000377578 | 184 |
| 166614 | DCAMKL2 | ENSP00000377578 | 207 |
| 166614 | DCAMKL2 | ENSP00000377578 | 302 |
| 166614 | DCAMKL2 | ENSP00000377578 | 397 |
| 166614 | DCAMKL2 | ENSP00000377578 | 608 |
| 1632 | DCI | ENSP00000301729 | 61 |
| 2145 | EZH1 | ENSP00000264646 | 140 |
| 2145 | EZH1 | ENSP00000264646 | 201 |
| 2145 | EZH1 | ENSP00000264646 | 254 |
| 2145 | EZH1 | ENSP00000264646 | 506 |
| 2145 | EZH1 | ENSP00000264646 | 714 |
| 6184 | RPN1 | ENSP00000296255 | 89 |
| 6184 | RPN1 | ENSP00000296255 | 465 |
| 10392 | CARD4 | ENSP00000222823 | 70 |
| 10392 | CARD4 | ENSP00000222823 | 142 |
| 10392 | CARD4 | ENSP00000222823 | 208 |
| 10392 | CARD4 | ENSP00000222823 | 830 |
| 10392 | CARD4 | ENSP00000222823 | 937 |
| 10392 | CARD4 | ENSP00000222823 | 942 |
| 10392 | CARD4 | ENSP00000222823 | 948 |
| 1807 | DPYS | ENSP00000276651 | 57 |
| 1807 | DPYS | ENSP00000276651 | 143 |
| 1807 | DPYS | ENSP00000276651 | 205 |
| 1807 | DPYS | ENSP00000276651 | 461 |
| 1621 | DBH | ENSP00000376776 | 248 |
| 1621 | DBH | ENSP00000376776 | 286 |
| 1621 | DBH | ENSP00000376776 | 393 |
| 64794 | DDX31 | ENSP00000361228 | 345 |
| 64794 | DDX31 | ENSP00000361228 | 350 |
| 2542 | SLC37A4 | ENSP00000350203 | 206 |
| 2542 | SLC37A4 | ENSP00000350203 | 211 |
| 7020 | TFAP2A | ENSP00000368933 | 2 |
| 7020 | TFAP2A | ENSP00000368933 | 6 |
| 7020 | TFAP2A | ENSP00000368933 | 12 |
| 51203 | NUSAP1 | ENSP00000260359 | 44 |
| 51203 | NUSAP1 | ENSP00000260359 | 132 |
| 51203 | NUSAP1 | ENSP00000260359 | 171 |
| 51203 | NUSAP1 | ENSP00000260359 | 220 |
| 51203 | NUSAP1 | ENSP00000260359 | 316 |
| 7597 | ZBTB25 | ENSP00000378204 | 204 |
| 27342 | RABGEF1 | ENSP00000370208 | 4 |
| 27342 | RABGEF1 | ENSP00000370208 | 273 |
| 27342 | RABGEF1 | ENSP00000370208 | 274 |
| 27342 | RABGEF1 | ENSP00000370208 | 321 |
| 27342 | RABGEF1 | ENSP00000370208 | 346 |
| 7205 | TRIP6 | ENSP00000200457 | 10 |
| 7205 | TRIP6 | ENSP00000200457 | 224 |
| 79651 | RHBDL6 | ENSP00000374410 | 6 |
| 79651 | RHBDL6 | ENSP00000374410 | 52 |
| 79651 | RHBDL6 | ENSP00000374410 | 443 |
| 79651 | RHBDL6 | ENSP00000374410 | 657 |
| 79651 | RHBDL6 | ENSP00000374410 | 772 |
| 1027 | CDKN1B | ENSP00000379629 | 73 |
| 1027 | CDKN1B | ENSP00000379629 | 134 |
| 1027 | CDKN1B | ENSP00000379629 | 172 |
| 23769 | FLRT1 | ENSP00000246841 | 155 |
| 23769 | FLRT1 | ENSP00000246841 | 489 |
| 2801 | GOLGA2 | ENSP00000342692 | 33 |
| 2801 | GOLGA2 | ENSP00000342692 | 218 |
| 2801 | GOLGA2 | ENSP00000342692 | 570 |
| 9550 | ATP6V1G1 | ENSP00000363162 | 34 |
| 10768 | AHCYL1 | ENSP00000377238 | 17 |
| 10768 | AHCYL1 | ENSP00000377238 | 147 |
| 10539 | TXNL2 | ENSP00000357633 | 67 |
| 10539 | TXNL2 | ENSP00000357633 | 231 |
| 10539 | TXNL2 | ENSP00000357633 | 253 |
| 10539 | TXNL2 | ENSP00000357633 | 308 |
| 8623 | ASMTL | ENSP00000370718 | 259 |
| 8623 | ASMTL | ENSP00000370718 | 314 |
| 8623 | ASMTL | ENSP00000370718 | 409 |
| 8623 | ASMTL | ENSP00000370718 | 563 |
| 56143 | PCDHA5 | ENSP00000367366 | 123 |
| 56143 | PCDHA5 | ENSP00000367366 | 651 |
| 414301 | DDI1 | ENSP00000302805 | 213 |
| 7278 | TUBA2 | ENSP00000382982 | 40 |
| 7278 | TUBA2 | ENSP00000382982 | 401 |
| 55250 | STATIP1 | ENSP00000316051 | 723 |
| 9181 | ARHGEF2 | ENSP00000357298 | 236 |
| 9181 | ARHGEF2 | ENSP00000357298 | 439 |
| 9181 | ARHGEF2 | ENSP00000357298 | 595 |
| 9181 | ARHGEF2 | ENSP00000357298 | 800 |
| 6871 | TADA2L | ENSP00000377915 | 14 |
| 6871 | TADA2L | ENSP00000377915 | 235 |
| 84324 | CIP29 | ENSP00000337632 | 28 |
| 84324 | CIP29 | ENSP00000337632 | 73 |
| 10783 | NEK6 | ENSP00000377749 | 141 |
| 23729 | CARKL | ENSP00000225519 | 100 |
| 9862 | THRAP4 | ENSP00000377684 | 2 |
| 9862 | THRAP4 | ENSP00000377684 | 7 |
| 9862 | THRAP4 | ENSP00000377684 | 29 |
| 9862 | THRAP4 | ENSP00000377684 | 156 |
| 9862 | THRAP4 | ENSP00000377684 | 182 |
| 9862 | THRAP4 | ENSP00000377684 | 328 |
| 9862 | THRAP4 | ENSP00000377684 | 386 |
| 9862 | THRAP4 | ENSP00000377684 | 984 |
| 79048 | SECISBP2 | ENSP00000364965 | 207 |
| 79048 | SECISBP2 | ENSP00000364965 | 527 |
| 79048 | SECISBP2 | ENSP00000364965 | 544 |
| 79048 | SECISBP2 | ENSP00000364965 | 700 |
| 648 | PCGF4 | ENSP00000365909 | 73 |
| 648 | PCGF4 | ENSP00000365909 | 88 |
| 84167 | FLJ21742 | ENSP00000221671 | 6 |
| 84167 | FLJ21742 | ENSP00000221671 | 65 |
| 84167 | FLJ21742 | ENSP00000221671 | 129 |
| 84167 | FLJ21742 | ENSP00000221671 | 149 |
| 84167 | FLJ21742 | ENSP00000221671 | 192 |
| 84167 | FLJ21742 | ENSP00000221671 | 529 |
| 9686 | VGLL4 | ENSP00000384705 | 62 |
| 55758 | RCOR3 | ENSP00000355972 | 42 |
| 55758 | RCOR3 | ENSP00000355972 | 74 |
| 55758 | RCOR3 | ENSP00000355972 | 267 |
| 55758 | RCOR3 | ENSP00000355972 | 304 |
| 55758 | RCOR3 | ENSP00000355972 | 325 |
| 5082 | PDCL | ENSP00000377826 | 114 |
| 6195 | RPS6KA1 | ENSP00000383967 | 44 |
| 6195 | RPS6KA1 | ENSP00000383967 | 54 |
| 6195 | RPS6KA1 | ENSP00000383967 | 198 |
| 6195 | RPS6KA1 | ENSP00000383967 | 349 |
| 6195 | RPS6KA1 | ENSP00000383967 | 621 |
| 6195 | RPS6KA1 | ENSP00000383967 | 642 |
| 6195 | RPS6KA1 | ENSP00000383967 | 697 |
| 10133 | OPTN | ENSP00000368022 | 196 |
| 10133 | OPTN | ENSP00000368022 | 223 |
| 10133 | OPTN | ENSP00000368022 | 246 |
| 10133 | OPTN | ENSP00000368022 | 267 |
| 10133 | OPTN | ENSP00000368022 | 333 |
| 10133 | OPTN | ENSP00000368022 | 340 |
| 10133 | OPTN | ENSP00000368022 | 378 |
| 10133 | OPTN | ENSP00000368022 | 429 |
| 26271 | FBXO5 | ENSP00000356210 | 2 |
| 26271 | FBXO5 | ENSP00000356210 | 18 |
| 26271 | FBXO5 | ENSP00000356210 | 237 |
| 26271 | FBXO5 | ENSP00000356210 | 243 |
| 3500 | IGHG1 | ENSP00000374991 | 157 |
| 2232 | FDXR | ENSP00000293195 | 70 |
| 3326 | HSPCB | ENSP00000360709 | 53 |
| 3326 | HSPCB | ENSP00000360709 | 148 |
| 3326 | HSPCB | ENSP00000360709 | 275 |
| 6122 | RPL3 | ENSP00000385762 | 231 |
| 6122 | RPL3 | ENSP00000385762 | 245 |
| 6122 | RPL3 | ENSP00000385762 | 260 |
| 9325 | TRIP4 | ENSP00000261884 | 70 |
| 9325 | TRIP4 | ENSP00000261884 | 128 |
| 9325 | TRIP4 | ENSP00000261884 | 256 |
| 9325 | TRIP4 | ENSP00000261884 | 402 |
| 9325 | TRIP4 | ENSP00000261884 | 576 |
| 51552 | RAB14 | ENSP00000362946 | 35 |
| 51552 | RAB14 | ENSP00000362946 | 140 |
| 4542 | MYO1F | ENSP00000344871 | 15 |
| 4542 | MYO1F | ENSP00000344871 | 776 |
| 4542 | MYO1F | ENSP00000344871 | 881 |
| 2026 | ENO2 | ENSP00000229277 | 54 |
| 2026 | ENO2 | ENSP00000229277 | 105 |
| 2026 | ENO2 | ENSP00000229277 | 197 |
| 2026 | ENO2 | ENSP00000229277 | 228 |
| 8835 | SOCS2 | ENSP00000376831 | 87 |
| 9684 | LRRC14 | ENSP00000292524 | 456 |
| 10635 | RAD51AP1 | ENSP00000309479 | 165 |
| 10635 | RAD51AP1 | ENSP00000309479 | 228 |
| 10635 | RAD51AP1 | ENSP00000309479 | 252 |
| 51478 | HSD17B7 | ENSP00000348664 | 3 |
| 515 | ATP5F1 | ENSP00000358736 | 177 |
| 84970 | C1orf94 | ENSP00000381121 | 67 |
| 284004 | FLJ23825 | ENSP00000337854 | 186 |
| 284004 | FLJ23825 | ENSP00000337854 | 337 |
| 10013 | HDAC6 | ENSP00000365804 | 138 |
| 10013 | HDAC6 | ENSP00000365804 | 553 |
| 51514 | DTL | ENSP00000355958 | 241 |
| 51514 | DTL | ENSP00000355958 | 397 |
| 51514 | DTL | ENSP00000355958 | 437 |
| 51514 | DTL | ENSP00000355958 | 600 |
| 51514 | DTL | ENSP00000355958 | 652 |
| 51514 | DTL | ENSP00000355958 | 669 |
| 51514 | DTL | ENSP00000355958 | 671 |
| 143630 | MGC20470 | ENSP00000369531 | 158 |
| 143630 | MGC20470 | ENSP00000369531 | 172 |
| 84733 | CBX2 | ENSP00000308750 | 131 |
| 84733 | CBX2 | ENSP00000308750 | 146 |
| 84733 | CBX2 | ENSP00000308750 | 153 |
| 84733 | CBX2 | ENSP00000308750 | 157 |
| 84733 | CBX2 | ENSP00000308750 | 191 |
| 5328 | PLAU | ENSP00000361847 | 129 |
| 5328 | PLAU | ENSP00000361847 | 217 |
| 5510 | PPP1R7 | ENSP00000385466 | 47 |
| 5510 | PPP1R7 | ENSP00000385466 | 91 |
| 55619 | DOCK10 | ENSP00000386392 | 368 |
| 55619 | DOCK10 | ENSP00000386392 | 560 |
| 55619 | DOCK10 | ENSP00000386392 | 603 |
| 55619 | DOCK10 | ENSP00000386392 | 705 |
| 55619 | DOCK10 | ENSP00000386392 | 1306 |
| 55619 | DOCK10 | ENSP00000386392 | 1441 |
| 55619 | DOCK10 | ENSP00000386392 | 1637 |
| 55619 | DOCK10 | ENSP00000386392 | 1640 |
| 55619 | DOCK10 | ENSP00000386392 | 1702 |
| 55619 | DOCK10 | ENSP00000386392 | 1928 |
| 55619 | DOCK10 | ENSP00000386392 | 2164 |
| 6251 | RSU1 | ENSP00000367145 | 224 |
| 6251 | RSU1 | ENSP00000367145 | 282 |
| 22885 | ABLIM3 | ENSP00000377875 | 2 |
| 22885 | ABLIM3 | ENSP00000377875 | 163 |
| 51163 | DBR1 | ENSP00000260803 | 511 |
| 3268 | HRBL | ENSP00000300176 | 7 |
| 3268 | HRBL | ENSP00000300176 | 173 |
| 10456 | HAX1 | ENSP00000329002 | 215 |
| 22895 | RPH3A | ENSP00000374036 | 642 |
| 149371 | EXOC8 | ENSP00000355605 | 266 |
| 149371 | EXOC8 | ENSP00000355605 | 545 |
| 149371 | EXOC8 | ENSP00000355605 | 698 |
| 55249 | YY1AP1 | ENSP00000385390 | 73 |
| 55249 | YY1AP1 | ENSP00000385390 | 234 |
| 55249 | YY1AP1 | ENSP00000385390 | 425 |
| 55249 | YY1AP1 | ENSP00000385390 | 638 |
| 55249 | YY1AP1 | ENSP00000385390 | 684 |
| 55249 | YY1AP1 | ENSP00000385390 | 716 |
| 200576 | PIP5K3 | ENSP00000384356 | 6 |
| 200576 | PIP5K3 | ENSP00000384356 | 94 |
| 55295 | FLJ11078 | ENSP00000300976 | 476 |
| 60496 | AASDHPPT | ENSP00000278618 | 6 |
| 60496 | AASDHPPT | ENSP00000278618 | 96 |
| 60496 | AASDHPPT | ENSP00000278618 | 185 |
| 54905 | CYP2W1 | ENSP00000344178 | 30 |
| 54905 | CYP2W1 | ENSP00000344178 | 363 |
| 147015 | MGC23280 | ENSP00000378361 | 319 |
| 29091 | STXBP6 | ENSP00000379928 | 4 |
| 29091 | STXBP6 | ENSP00000379928 | 66 |
| 85360 | SYDE1 | ENSP00000341489 | 89 |
| 85360 | SYDE1 | ENSP00000341489 | 169 |
| 85360 | SYDE1 | ENSP00000341489 | 444 |
| 10113 | PREB | ENSP00000384032 | 107 |
| 10113 | PREB | ENSP00000384032 | 126 |
| 10113 | PREB | ENSP00000384032 | 127 |
| 10113 | PREB | ENSP00000384032 | 188 |
| 10113 | PREB | ENSP00000384032 | 297 |
| 6525 | SMTN | ENSP00000385265 | 50 |
| 6525 | SMTN | ENSP00000385265 | 131 |
| 140735 | Dlc2 | ENSP00000240343 | 5 |
| 51177 | CKIP-1 | ENSP00000358122 | 152 |
| 51177 | CKIP-1 | ENSP00000358122 | 187 |
| 10391 | CORO2B | ENSP00000378836 | 5 |
| 10391 | CORO2B | ENSP00000378836 | 94 |
| 55166 | C6orf139 | ENSP00000360243 | 4 |
| 55166 | C6orf139 | ENSP00000360243 | 130 |
| 4675 | NAP1L3 | ENSP00000362171 | 135 |
| 4675 | NAP1L3 | ENSP00000362171 | 203 |
| 4675 | NAP1L3 | ENSP00000362171 | 217 |
| 4675 | NAP1L3 | ENSP00000362171 | 231 |
| 4675 | NAP1L3 | ENSP00000362171 | 304 |
| 4675 | NAP1L3 | ENSP00000362171 | 453 |
| 9751 | SNPH | ENSP00000386414 | 290 |
| 11274 | USP18 | ENSP00000215794 | 3 |
| 11274 | USP18 | ENSP00000215794 | 152 |
| 11274 | USP18 | ENSP00000215794 | 240 |
| 11274 | USP18 | ENSP00000215794 | 288 |
| 201456 | FBXO15 | ENSP00000269500 | 85 |
| 144699 | FBXL14 | ENSP00000344855 | 42 |
| 7086 | TKT | ENSP00000296289 | 6 |
| 7086 | TKT | ENSP00000296289 | 241 |
| 7086 | TKT | ENSP00000296289 | 543 |
| 1267 | CNP | ENSP00000377470 | 261 |
| 23759 | PPIL2 | ENSP00000381812 | 3 |
| 23759 | PPIL2 | ENSP00000381812 | 180 |
| 23759 | PPIL2 | ENSP00000381812 | 450 |
| 4208 | MEF2C | NP_002388.2 | 391 |
| 51310 | SLC22A17 | ENSP00000380437 | 306 |
| 57697 | FANCM | ENSP00000267430 | 136 |
| 57697 | FANCM | ENSP00000267430 | 178 |
| 57697 | FANCM | ENSP00000267430 | 209 |
| 57697 | FANCM | ENSP00000267430 | 530 |
| 57697 | FANCM | ENSP00000267430 | 1155 |
| 57697 | FANCM | ENSP00000267430 | 1316 |
| 57697 | FANCM | ENSP00000267430 | 1428 |
| 57697 | FANCM | ENSP00000267430 | 1510 |
| 55718 | POLR3E | ENSP00000352140 | 498 |
| 79085 | SLC25A23 | ENSP00000334537 | 177 |
| 2167 | FABP4 | ENSP00000256104 | 22 |
| 2167 | FABP4 | ENSP00000256104 | 53 |
| 129807 | NEU4 | ENSP00000385149 | 476 |
| 3854 | KRT6B | ENSP00000252252 | 338 |
| 3854 | KRT6B | ENSP00000252252 | 436 |
| 220965 | FAM13C1 | ENSP00000362974 | 324 |
| 220965 | FAM13C1 | ENSP00000362974 | 396 |
| 220965 | FAM13C1 | ENSP00000362974 | 427 |
| 91133 | L3MBTL4 | ENSP00000382976 | 2 |
| 91133 | L3MBTL4 | ENSP00000382976 | 159 |
| 91133 | L3MBTL4 | ENSP00000382976 | 289 |
| 91133 | L3MBTL4 | ENSP00000382976 | 301 |
| 91133 | L3MBTL4 | ENSP00000382976 | 413 |
| 5316 | PKNOX1 | ENSP00000291547 | 260 |
| 5316 | PKNOX1 | ENSP00000291547 | 293 |
| 8773 | SNAP23 | ENSP00000380327 | 40 |
| 8773 | SNAP23 | ENSP00000380327 | 132 |
| 81570 | CLPB | ENSP00000340385 | 253 |
| 81570 | CLPB | ENSP00000340385 | 357 |
| 81570 | CLPB | ENSP00000340385 | 392 |
| 81570 | CLPB | ENSP00000340385 | 628 |
| 8568 | D21S2056E | ENSP00000383237 | 68 |
| 8568 | D21S2056E | ENSP00000383237 | 330 |
| 54682 | MANSC1 | ENSP00000379638 | 77 |
| 54682 | MANSC1 | ENSP00000379638 | 383 |
| 8507 | ENC1 | ENSP00000306356 | 309 |
| 8877 | SPHK1 | ENSP00000376285 | 26 |
| 5713 | PSMD7 | ENSP00000219313 | 103 |
| 5713 | PSMD7 | ENSP00000219313 | 127 |
| 5713 | PSMD7 | ENSP00000219313 | 180 |
| 5713 | PSMD7 | ENSP00000219313 | 288 |
| 5713 | PSMD7 | ENSP00000219313 | 293 |
| 5713 | PSMD7 | ENSP00000219313 | 316 |
| 26256 | CABYR | ENSP00000382405 | 66 |
| 26256 | CABYR | ENSP00000382405 | 207 |
| 26256 | CABYR | ENSP00000382405 | 415 |
| 26256 | CABYR | ENSP00000382405 | 448 |
| 23062 | GGA2 | ENSP00000311962 | 215 |
| 23062 | GGA2 | ENSP00000311962 | 577 |
| 23062 | GGA2 | ENSP00000311962 | 602 |
| 112476 | LOC112476 | ENSP00000351608 | 111 |
| 23059 | CLUAP1 | ENSP00000344392 | 85 |
| 23059 | CLUAP1 | ENSP00000344392 | 138 |
| 23059 | CLUAP1 | ENSP00000344392 | 303 |
| 23059 | CLUAP1 | ENSP00000344392 | 402 |
| 55722 | Cep72 | ENSP00000264935 | 115 |
| 55722 | Cep72 | ENSP00000264935 | 145 |
| 9904 | RBM19 | ENSP00000376344 | 34 |
| 9904 | RBM19 | ENSP00000376344 | 50 |
| 9904 | RBM19 | ENSP00000376344 | 347 |
| 9904 | RBM19 | ENSP00000376344 | 481 |
| 9904 | RBM19 | ENSP00000376344 | 482 |
| 9904 | RBM19 | ENSP00000376344 | 646 |
| 9904 | RBM19 | ENSP00000376344 | 712 |
| 9904 | RBM19 | ENSP00000376344 | 783 |
| 9904 | RBM19 | ENSP00000376344 | 859 |
| 9904 | RBM19 | ENSP00000376344 | 866 |
| 55701 | FLJ10357 | ENSP00000298694 | 122 |
| 55701 | FLJ10357 | ENSP00000298694 | 377 |
| 55701 | FLJ10357 | ENSP00000298694 | 405 |
| 55701 | FLJ10357 | ENSP00000298694 | 440 |
| 55701 | FLJ10357 | ENSP00000298694 | 717 |
| 55701 | FLJ10357 | ENSP00000298694 | 1178 |
| 55701 | FLJ10357 | ENSP00000298694 | 1360 |
| 80174 | DRF1 | ENSP00000377178 | 6 |
| 80174 | DRF1 | ENSP00000377178 | 135 |
| 80174 | DRF1 | ENSP00000377178 | 222 |
| 80174 | DRF1 | ENSP00000377178 | 232 |
| 80174 | DRF1 | ENSP00000377178 | 239 |
| 80174 | DRF1 | ENSP00000377178 | 389 |
| 10227 | TETRAN | ENSP00000347619 | 230 |
| 10227 | TETRAN | ENSP00000347619 | 310 |
| 10227 | TETRAN | ENSP00000347619 | 453 |
| 4144 | MAT2A | ENSP00000386436 | 88 |
| 11133 | KPTN | ENSP00000337850 | 103 |
| 4841 | NONO | ENSP00000362963 | 11 |
| 4841 | NONO | ENSP00000362963 | 60 |
| 4841 | NONO | ENSP00000362963 | 338 |
| 11319 | HSGT1 | ENSP00000362070 | 63 |
| 11319 | HSGT1 | ENSP00000362070 | 80 |
| 11319 | HSGT1 | ENSP00000362070 | 222 |
| 11319 | HSGT1 | ENSP00000362070 | 401 |
| 11319 | HSGT1 | ENSP00000362070 | 402 |
| 11319 | HSGT1 | ENSP00000362070 | 434 |
| 55040 | EPN3 | ENSP00000268933 | 464 |
| 23378 | KIAA0409 | ENSP00000254605 | 219 |
| 23378 | KIAA0409 | ENSP00000254605 | 440 |
| 10922 | FASTK | ENSP00000352212 | 80 |
| 9112 | MTA1 | ENSP00000384180 | 243 |
| 9112 | MTA1 | ENSP00000384180 | 250 |
| 23291 | FBXW11 | ENSP00000377393 | 305 |
| 8099 | CDK2AP1 | ENSP00000261692 | 92 |
| 922 | CD5L | ENSP00000357156 | 173 |
| 922 | CD5L | ENSP00000357156 | 299 |
| 116224 | C9orf42 | ENSP00000366492 | 89 |
| 27161 | EIF2C2 | ENSP00000220592 | 62 |
| 27161 | EIF2C2 | ENSP00000220592 | 91 |
| 27161 | EIF2C2 | ENSP00000220592 | 354 |
| 27161 | EIF2C2 | ENSP00000220592 | 402 |
| 27161 | EIF2C2 | ENSP00000220592 | 533 |
| 27161 | EIF2C2 | ENSP00000220592 | 693 |
| 27161 | EIF2C2 | ENSP00000220592 | 844 |
| 1160 | CKMT2 | ENSP00000254035 | 230 |
| 1160 | CKMT2 | ENSP00000254035 | 335 |
| 4007 | LMO6 | ENSP00000365487 | 101 |
| 4007 | LMO6 | ENSP00000365487 | 169 |
| 92703 | TMEM183A | ENSP00000356211 | 68 |
| 92703 | TMEM183A | ENSP00000356211 | 270 |
| 7251 | TSG101 | ENSP00000349721 | 199 |
| 51322 | WAC | ENSP00000364797 | 334 |
| 51322 | WAC | ENSP00000364797 | 351 |
| 51322 | WAC | ENSP00000364797 | 417 |
| 51322 | WAC | ENSP00000364797 | 429 |
| 115509 | ZNF689 | ENSP00000287461 | 30 |
| 115509 | ZNF689 | ENSP00000287461 | 137 |
| 56474 | CTPS2 | ENSP00000369555 | 19 |
| 6175 | RPLP0 | ENSP00000376299 | 146 |
| 6175 | RPLP0 | ENSP00000376299 | 297 |
| 9094 | UNC119 | ENSP00000337040 | 57 |
| 5859 | QARS | ENSP00000307567 | 80 |
| 5859 | QARS | ENSP00000307567 | 166 |
| 5859 | QARS | ENSP00000307567 | 205 |
| 5859 | QARS | ENSP00000307567 | 292 |
| 5859 | QARS | ENSP00000307567 | 394 |
| 5859 | QARS | ENSP00000307567 | 740 |
| 6187 | RPS2 | ENSP00000341885 | 74 |
| 23154 | NCDN | ENSP00000381017 | 663 |
